# Intermittent hypoxia induces Th17/Treg imbalance in a murine model of obstructive sleep apnea

**DOI:** 10.1371/journal.pone.0305230

**Published:** 2024-06-24

**Authors:** Do-Yang Park, Chang-Hoon Kim, Da-Young Park, Hyun Jun Kim, Hyung-Ju Cho

**Affiliations:** 1 Department of Otolaryngology, Ajou University School of Medicine, Suwon, Republic of Korea; 2 Department of Medicine, Graduate School, Yonsei University, Seoul, Republic of Korea; 3 Department of Otorhinolaryngology, Yonsei University College of Medicine, Seoul, Republic of Korea; 4 The Airway Mucus Institute, Yonsei University College of Medicine, Seoul, Republic of Korea; University of South Florida, UNITED STATES

## Abstract

Obstructive sleep apnea (OSA) is characterized by cyclic normoxic and hypoxic conditions (intermittent hypoxia, IH) induced by the repeated closure of the upper-airway respiratory tract. As a pathomechanism of OSA, IH results in various comorbidities via chronic inflammation and related pathways. However, the role of other inflammatory cells, such as lymphocytes, has not been well-explored. This study aimed to examine the effects of IH on the distribution and balance of T cell subsets and other related cytokines, and mechanisms in the immune system. We modified OSA mouse model (male C57BL/6N male) using our customized chamber that controls specific sleep and oxygenic cycles. To induce hypoxia, the IH group was repeatedly exposed to 5% O_2_ and 21% O_2_ lasting for 120 s each for 7 h daily for 4 weeks. Mice were then subjected to a recovery period of 4 weeks, in which IH stimulation was ceased. T cells and related cytokines were analyzed using flow cytometry and immunohistochemistry. Compared with the control group, the IH group had significantly lower levels of CD4^+^CD25^+^Foxp3^+^ regulatory T cells but higher levels of Th 17, IL-4, HIF-1, and inflammatory cytokines. After the recovery period, these altered changes in the immune cells were recovered, and we found no significant difference in their levels between the control and recovery groups. This study revealed that the Th17/Treg ratio is increased by intermittent hypoxia, and this imbalance can explain immune-related diseases, including recently reported allergies, autoimmune, and even cancer diseases, arising from OSA.

## Introduction

Obstructive sleep apnea (OSA) is a sleep-related breathing disorder characterized by a complete or partial collapse of the upper airway during sleep and affects 3–9% of the general population [1–3]. It is associated with mild to fatal complications, such as resistant hypertension, cardiovascular diseases, neurological diseases, and various types of mortality [4, 5]. In addition, in America, moderate to severe sleep apnea in middle-aged adults results in wages and productivity losses, motor-vehicle collisions, and fatalities, high medical expenses [6]. Moreover, recent studies have proposed that the cumulative burden of chronic and low-grade systemic inflammation serves as the underlying mechanism for the various complications associated with OSA [7]. Furthermore, there are reports indicating that chronic systemic inflammation can lead to alterations in the distribution and ratio of individual immune cells [8].

Sleep is characterized by its interaction with the endocrine and autonomic nervous systems, both of which play crucial roles in regulating various physiological processes. Moreover, normal sleep is essential for maintaining optimal immune function. Recent studies have documented that sleep disturbances lead to alterations in the distribution and functionality of immune cells, with a particular focus on T cells [9]. In particular, studies have indicated a shift in the ratio of T helper 1 (Th1) and Th2 cells towards Th2 dominance in patients with obstructive sleep apnea syndrome (OSAS) [10]. Such Th2 dominance can lead to an excessive production of anti-inflammatory cytokines and antibodies, potentially compromising the body’s capability to eradicate self-reactive immune cells or pathogens, consequently predisposing to autoimmunity. Furthermore, Th2 dominance may disrupt the function or differentiation of regulatory T cells (Tregs), resulting in impaired suppression of autoreactive T cells and exacerbating autoimmune diseases. It has been reported that this mechanism could potentially exacerbate autoimmune diseases in patients with OSAS [11].

The balance between Th17 and Treg also plays a significant role in autoimmunity and inflammation, with imbalances in the Th17/Treg ratio reported in various chronic inflammatory and immunologic diseases [12, 13]. Recent clinical studies have demonstrated a notable alteration in T cell distribution among pediatric patients with OSA, characterized by a decrease in Tregs and an increase in Th17 cells. Moreover, this imbalance becomes more pronounced with the severity of OSA [14]. These alterations in T cell distribution may influence the expression or severity of immunologic diseases, including autoimmune and allergic diseases. Notably, there appears to be a high prevalence of allergic rhinitis among patients with obstructive sleep apnea [15]. While it has been traditionally assumed that airway narrowing due to allergic disease exacerbates obstructive sleep apnea, it is also plausible that changes in T cell distribution among obstructive sleep apnea patients could negatively impact the expression or severity of allergic diseases. This suggests the potential for a vicious cycle wherein alterations in T cell profiles among obstructive sleep apnea patients and airway narrowing resulting from allergic diseases mutually exacerbate each other. The complete or partial collapse of the upper airway in patients with OSA results in repeated episodes of normoxia and hypoxia in the body. We hypothesized that this repetitive pattern, known as intermittent hypoxia, may have an impact on T cell distribution.

Various *in vitro* studies have investigated the effects of sleep apnea [16–18]. However, rapidly changing the oxygen partial pressure in the culture media to maintain levels similar to those found in the lungs of patients with sleep apnea is challenging in *in vitro* studies. In this study, we have adapted the previous OSA mouse model to suit our research requirements and successfully established our OSA mouse model, which reflects the objectives of our experiments and mimics the status of OSA [19–21]. Using this model, we investigated the impact of intermittent hypoxia on T cell distribution, balance, and associated cytokines.

## Material and methods

### Experimental animals

Four-week-old specific pathogen-free (SPF) male C57BL/6N mice, each weighing 14–16 g, were used. The animals were housed in an air-conditioned room maintained at 25 ± 2°C and 50–60% humidity. All the mice were fed with free access to water and food. All experiments were carried out with the approval of the Institutional Animal Care and Use Committee of the Ajou University Medical Center (approval number 2017–0035).

### Obstructive sleep apnea mouse model

**Fig 1:**
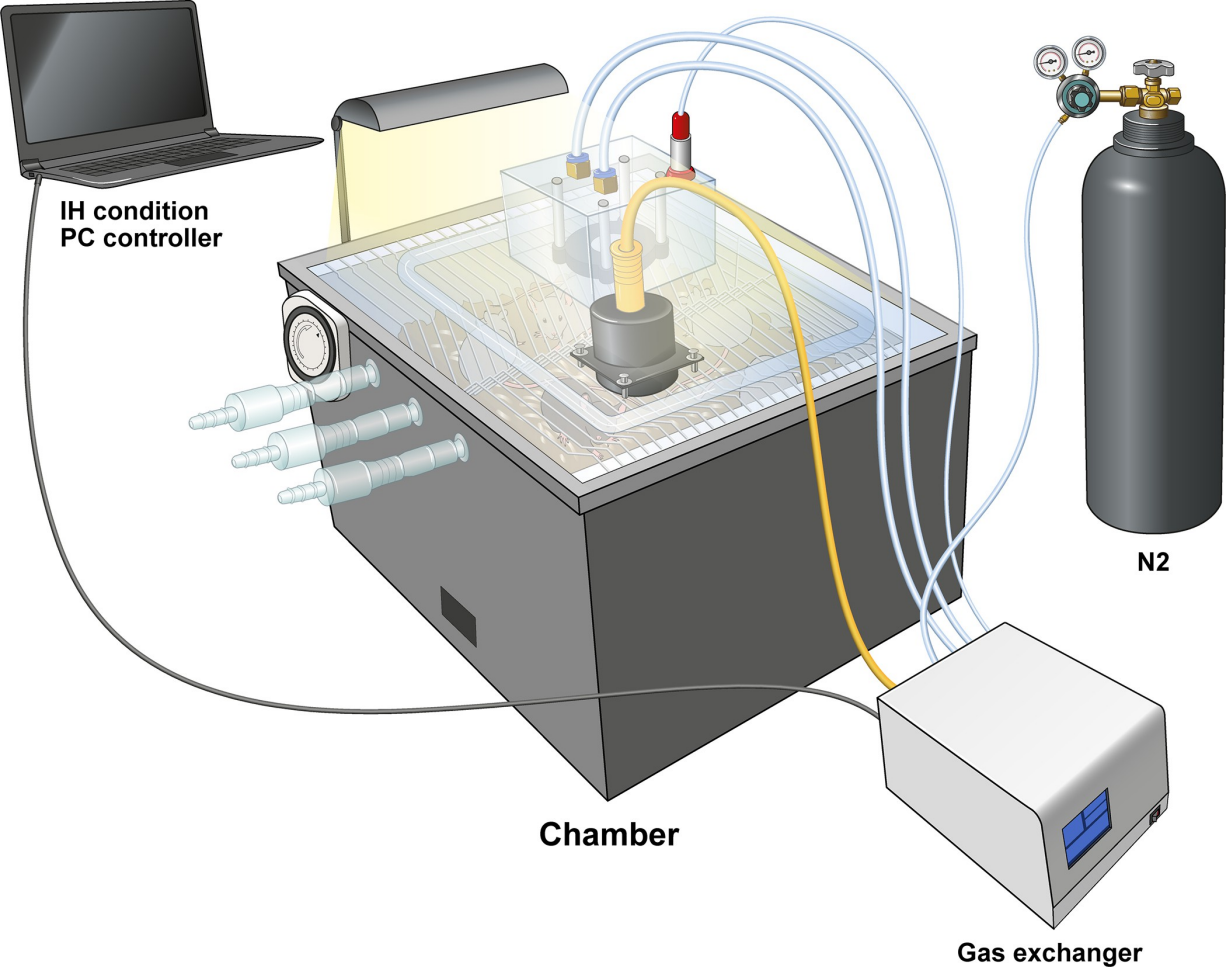
Customized intermittent hypoxia chamber to establish intermittent hypoxia in a murine model of OSA. (A) Customized chamber with gas pump, (B) gas exchanger, (C) PC controller of intermittent hypoxia, and (D) nitrogen gas source. The capacities of the nitrogen and oxygen gas pumps were designed to rapidly alter the air composition in the chamber from 21% O_2_ (room air conditions) to 1% O_2_ within a minute. This corresponds to a maximum of 30 apnea conditions per hour.

**Fig 2:**
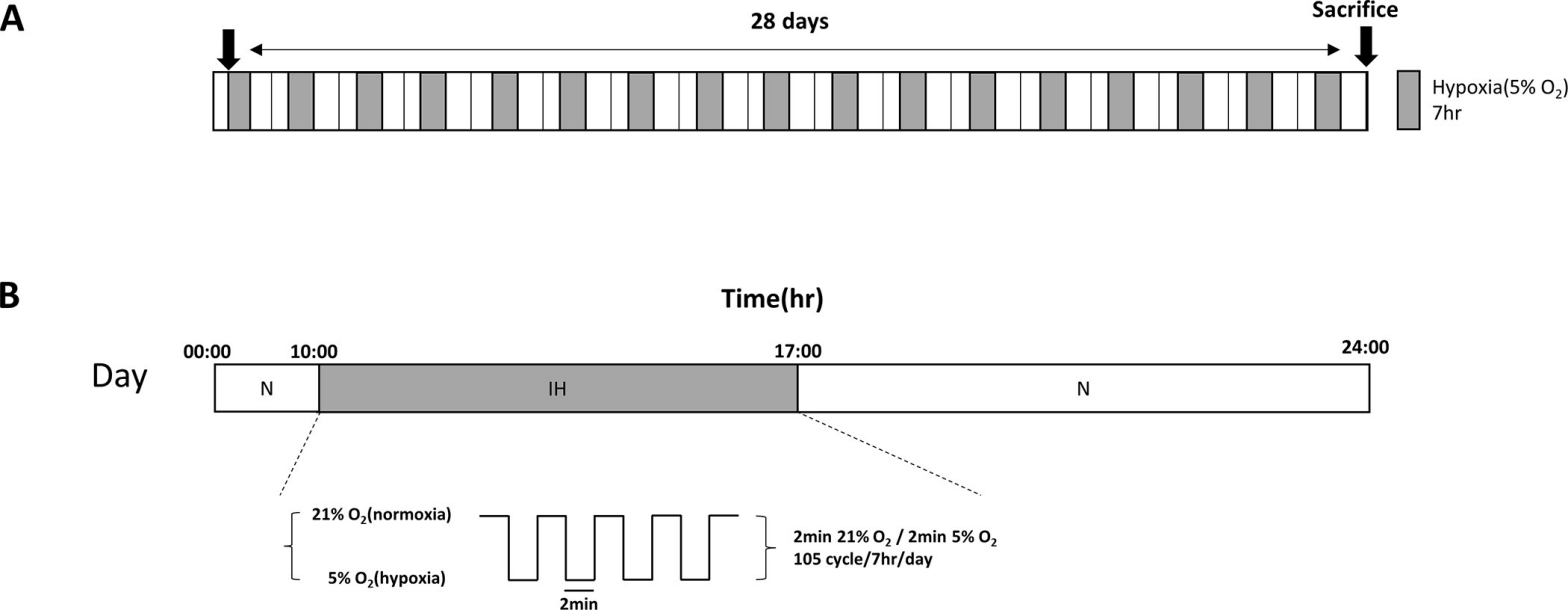
Experimental timeline to study the effect of nasal exposure of mice to intermittent hypoxia. Graphical representation of the intermittent hypoxia (IH) protocol. (A) IH protocol was conducted for 28 days during the 7-h light phase. (B) The IH groups were exposed to intermittent hypoxia for 7 h per day. During IH, the fractional inspired O_2_ was reduced from room air levels to approximately 5% within 2 min, followed by re-oxygenation to room air levels within the subsequent 2-min period.

C57BL/6N mice were acclimated for one week after purchase. The mice were divided into two groups: normoxia (N group, *n* = 10) and intermittent hypoxia (IH group, *n* = 10). To minimize the stress in mice, the experiment was conducted with a maximum of 5 mice per chamber at a time. A customized intermittent hypoxia chamber was applied to the IH group mice to establish the OSA mouse model (Fig 1). The IH group was exposed to intermittent hypoxia from 10 am to 5 pm daily. Using an infusion pump of N_2_ into the chambers during daytime, intermittent hypoxia conditions consisted of two-min cycles of 5% O_2_ inside chamber, followed by restoration to 21% O_2_ with a total of 15 cycles per hour (Fig 2A and 2B). Compared with this repetitive hypoxic condition, a schedule is applied 15 times per hour, based on the human apnea-hypopnea index, these repetitive normoxia and hypoxia conditions corresponded to moderately severe OSA in humans. After exposure to IH for 4 weeks, all mice were euthanized by cervical dislocation by a trained professional after isoflurane anesthesia.

### Measurement of arterial oxygen saturation

Arterial oxygen saturation (SpO_2_) was measured from 9 am to 10 pm using a mouse oxymeter (MouseOx) PhysioSuite® and MouseSTAT® (Kent Scientific, Torrington, CT, USA). On the other hand, in the control group, saturation was measured without intermittent hypoxia. Briefly, mice were gently restrained inside hypoxic or normoxic chambers. A collar clip (XS size) with a sensor probe (4 mm) was placed on the temporal regions of their tail. Only error code-free data analyzed using the MouseOx software were included in the analysis (STARR Life Science Corp., Allison Park, PA, USA).

### Obstructive sleep apnea recovery mouse model

To clarify the relationship between intermittent hypoxia and the altered distributions of T cell subsets, after 4 weeks of intermittent hypoxia, stimulation was ceased for 4 weeks to allow recovery. Mice were further divided into two groups: control rest (C-rest group, *n* = 6) and intermittent hypoxia rest group (IH-rest group, *n* = 6).

### Quantification of T cells using flow cytometry

Spleens were harvested at the end schedules of IH (4 weeks) or IH/rest (8 weeks) and make single-cell suspensions. Splenocytes were passed through a 70-μm cell strainer and washed with 10 mL of 1X PBS (+2% FBS). 1X lysis buffer (BD Biosciences, San Jose, CA, USA) was used to lyse red blood cells. After washing, cells (3x10^6^ cells/ml) were cultured with complete RPMI 1640 medium supplemented with RMA/ionomycin (50 μg/ml and 1ug/ml, respectively) and protein transport inhibitor at 37°C and 5% CO_2_ for 5 h. Cultured cells were collected, washed with complete 1X PBS (+2% FBS), surface-labeled with CD4-PerCP-cy5.5, and fixed and permeabilized using a fixation/permeabilization reagent (BD Biosciences, San Jose, CA, USA). Finally, cells were stained with IFN-*γ*-FITC (Th1), IL-4-APC (Th2), and IL-17-PE (Th17).

Splenocytes were aliquoted into tubes without PMA. They were stimulated with ionomycin, surface-labeled with CD4-FITC and CD25-APC, fixed, permeabilized, and stained with FoxP3-PE to analyze Treg.

Labeled cells were washed and analyzed using a FACSAria III flow cytometer (BD Biosciences) and the BD FACSDiva software. Staining was compared with that of the appropriately labeled isotype control antibody.

### Western blotting

Spleen tissues were harvested at the time of death for protein extraction. Tissues were homogenized in RIPA lysis buffer (Thermo Fisher Scientific, Waltham, MA, USA) with protease and phosphatase inhibitor cocktails, sonicated, and stored at −20°C until use. Each tissue homogenate (30 μg) was used for protein electrophoresis on 10% polyacrylamide gel according to the manufacturer’s instructions. Proteins were then transferred onto polyvinylidene difluoride (PVDF) membranes and incubated with HIF-1α, phosphor-p65, p65, Foxp3, and β-actin antibodies. The detection of the chemiluminescent antibodies was performed using a commercially available kit (HRP chemiluminescent substrate) and exposed to film on an X-ray film processer (JPI Healthcare Solutions Inc., Plainview, NY, USA). β-actin was used as the internal contol. Results were analyzed using ImageJ software and reported as the pixel intensity of a target protein relative to β-actin housekeeping proteins.

### Immunohistochemistry

Mice were decapitated, and heads were prepared. Paraffin blocks were stained with HIF-1α, Foxp3, p65, Phospho-p65, IL-6, and TNF-α. Immunostained slides of the nasal maxilloturbinate were observed under a light microscop (magnification 400×) and analyzed using ImageJ software. Data are reported as the pixel intensity of an immunostained area relative to the total epithelial area.

### Statistical analysis

Data are expressed as mean ± standard deviation and are representative of three independent experiments. Mann–Whitney U test (SPSS v17, Chicago, IL, USA) was used for statistical analysis. *P*-value < 0.05 was considered statistically significant.

## Results

### Establishment and validation of the OSA mouse model

To investigate the hypothesis regarding the impact of intermittent hypoxia on T cell distribution, we established a customized hypoxia chamber, drawing upon modifications made to existing intermittent hypoxia chambers utilized by researchers such as Gozal et al. [22–25]. It was large enough to accommodate a normal mouse cage (261 × 374.5 × 216.5 mm, w/d/h) to minimize animal stress. The inside of the chamber can be completely sealed off from the external environment, and the composition of the internal gases can be changed to the desired level with computer-controlled infusion pump. The capacities of the gas pumps were designed to change the air composition in the chamber from 21% O_2_ (normal room condition) to 1% O_2_ within approximately 1 min. This corresponds to a maximum of 30 apnea conditions per hour (1 min hypoxia and 1 min normoxia, two-min cycle, 30 times/h) when the O_2_ partial pressure is changed in the chamber with maximum speed, representative of severe obstructive sleep apnea in humans. The amount of light entering the chamber from the outside can be regulated with timer to minimize the effects of irregular circadian rhythm.

**Fig 3:**
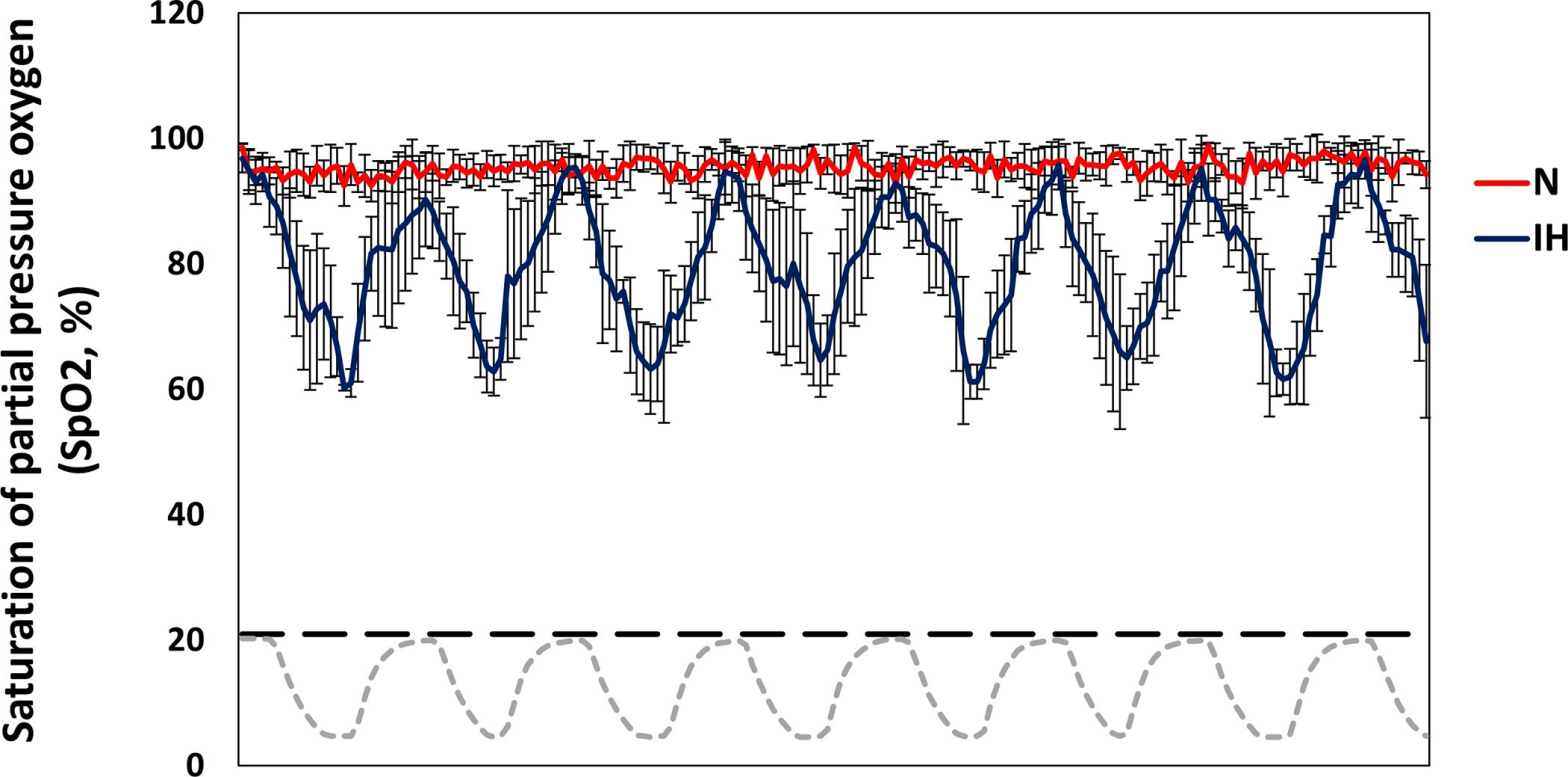
The fluctuation of oxygen saturation levels in the intermittent hypoxia mouse model and the control (normoxia). The data obtained by exposing to intermittent hypoxia and normoxia protocols for 30 minutes. Data are expressed as the mean ± S.E. (C57BL/6N mice, *n* = 5 per group). N, normoxia group. IH, intermittent hypoxia group.

The mean oxygen saturation levels of the N and IH groups were 95.39 ± 0.33% and 79.85 ± 1.84%, respectively, and the lowest oxygen saturation levels were 88.6% and 59.2%, respectively (Fig 3). Changes in oxygen partial pressure in the IH chamber were observed in accordance with oxygen saturation in the mice.

### Changes in T cell distribution

**Fig 4:**
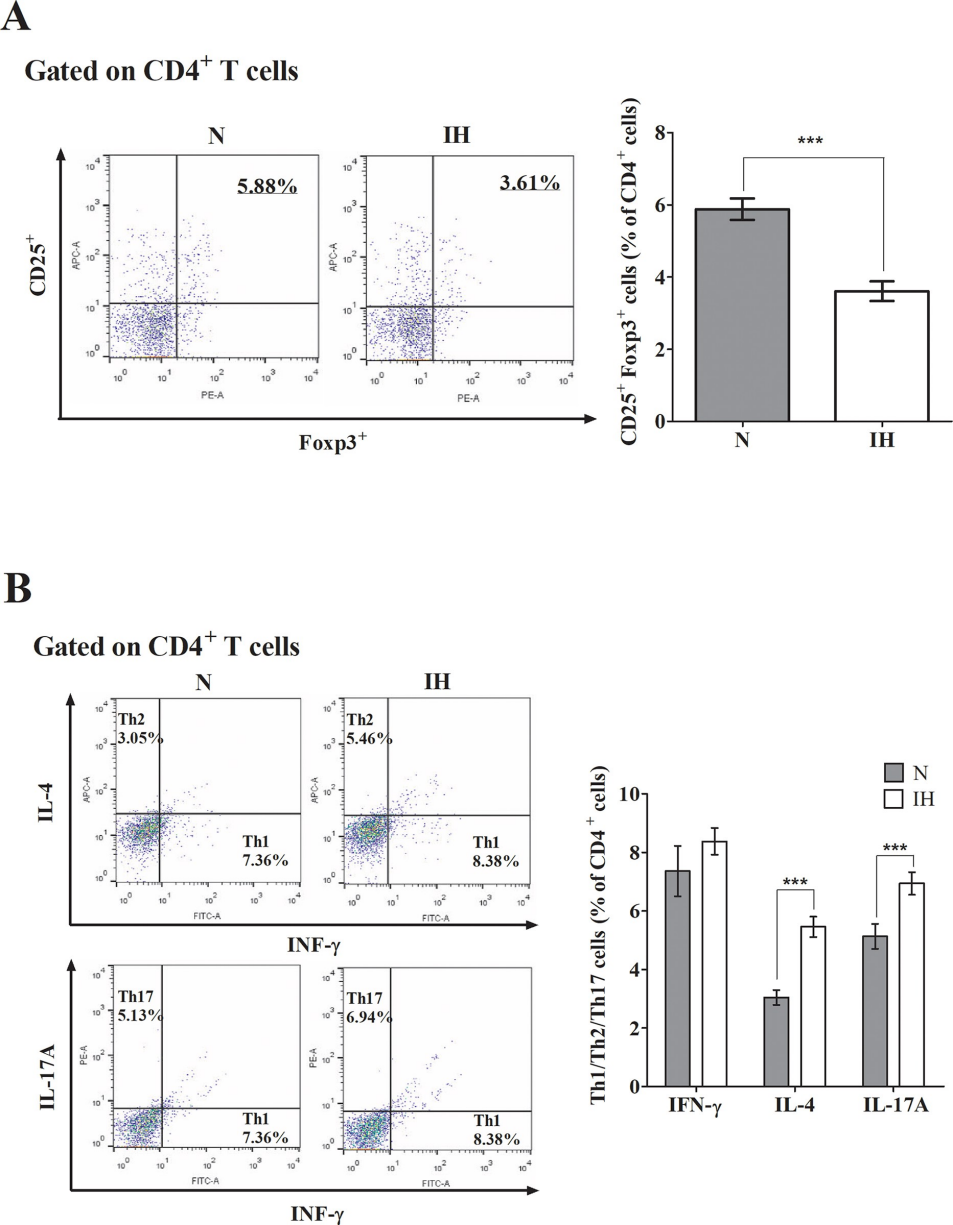
Frequency of regulatory T cells and cytokines Th1, Th2, and Th17 measured using flow cytometry in the OSA mouse model. (A) In the IH group, the distribution of decreased CD4 ^+^ CD25 ^+^ Foxp3 ^+^ regulatory T cells was statistically significant compared to the N group. Data are expressed as the mean ± S.E. (C57BL/6N mice, *n* = 10 per group). (B) IL-17A and IL-4 were increased significant compared to the N group. Data are expressed as the mean ± S.E. (C57BL/6N mice, *n* = 8 per group). N, normoxia group. IH, intermittent hypoxia group. The significant difference between groups were indicated by asterisks (*p < 0.05, **p < 0.01, ***p < 0.001, ****p <0.0001).

We assessed T cell distribution by analyzing the frequencies of CD4^+^CD25^+^Foxp3^+^ regulatory T cells as well as Th1, Th2, and Th17 subsets using FACS staining. The levels of CD4^+^, CD25^+^, and Foxp3^+^ regulatory T cells were significantly reduced in the IH group compared with the N group as observed by FACS staining (Fig 4). In contrast, those of IL-17A and IL-4 were significantly increased in the IH group compared with the N group. The level of IFN-γ was not significantly different between the two groups (Fig 4).

### Changes in Foxp3, NF-κB components, and inflammatory factors

**Fig 5:**
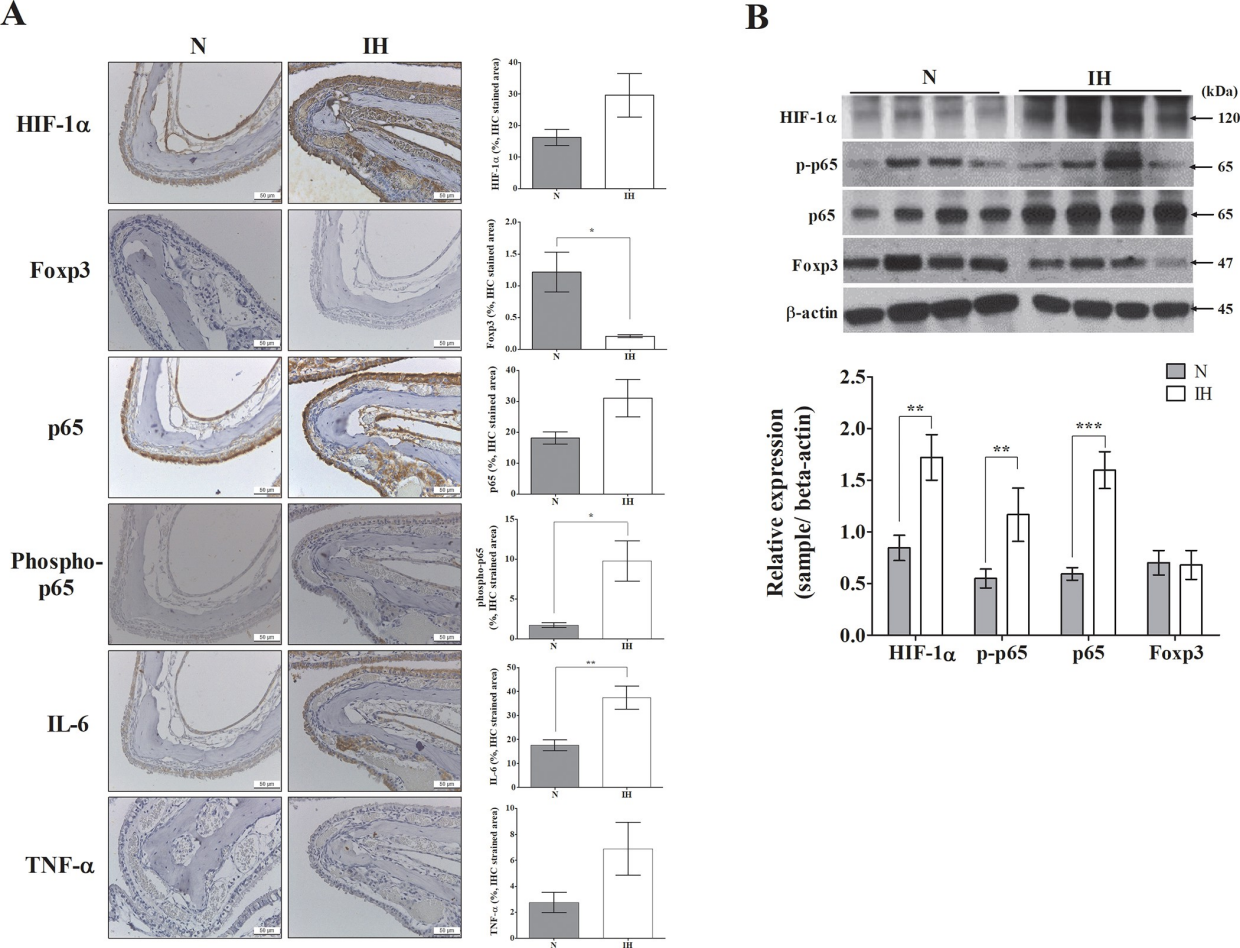
Histopathological analysis of nasal tissues and their quantification with western blotting in the OSA mouse model. (A) There were increase in HIF-1α, p65, phosphor-p65, IL-6 and TNF- α and a significant decrease in Foxp3 in the IH group compared to the N group. Data are expressed as the mean ± S.E. (C57BL/6N mice, *n* = 4 per group). (B) The expression of HIF-1α, phospho-65, and p65 increased while the expression of Foxp3 was decreased. Data are expressed as the mean ± S.E. (C57BL/6N mice, n = 10 per group). N, normoxia group. IH, Intermittent hypoxia group. The significant difference between groups were indicated by asterisks (*p < 0.05, **p < 0.01, ***p < 0.001, ****p <0.0001).

We elucidated the related mechanisms by analyzing cytokines associated with the results obtained from our FACS staining through western blot and immunohistochemistry analyses. From the results of western blotting, the expression levels of HIF-1α, p65, and phospho-p65 were significantly increased in the IH group, whereas that of Foxp3 was decreased but the change was not significant (Fig 5). Similarly, immunohistochemistry analysis confirmed the increased levels of HIF-1α, significantly decreased levels of Foxp3, increased activation of the NF-κB pathway (p65 and phospho-p65), and increased levels of inflammatory molecules in the nasal tissues of the IH group (Fig 5).

### Differentiation of CD4+CD25+Foxp3+ T cells and Th1/Th2/Th17 cytokine following hypoxia recovery

**Fig 6:**
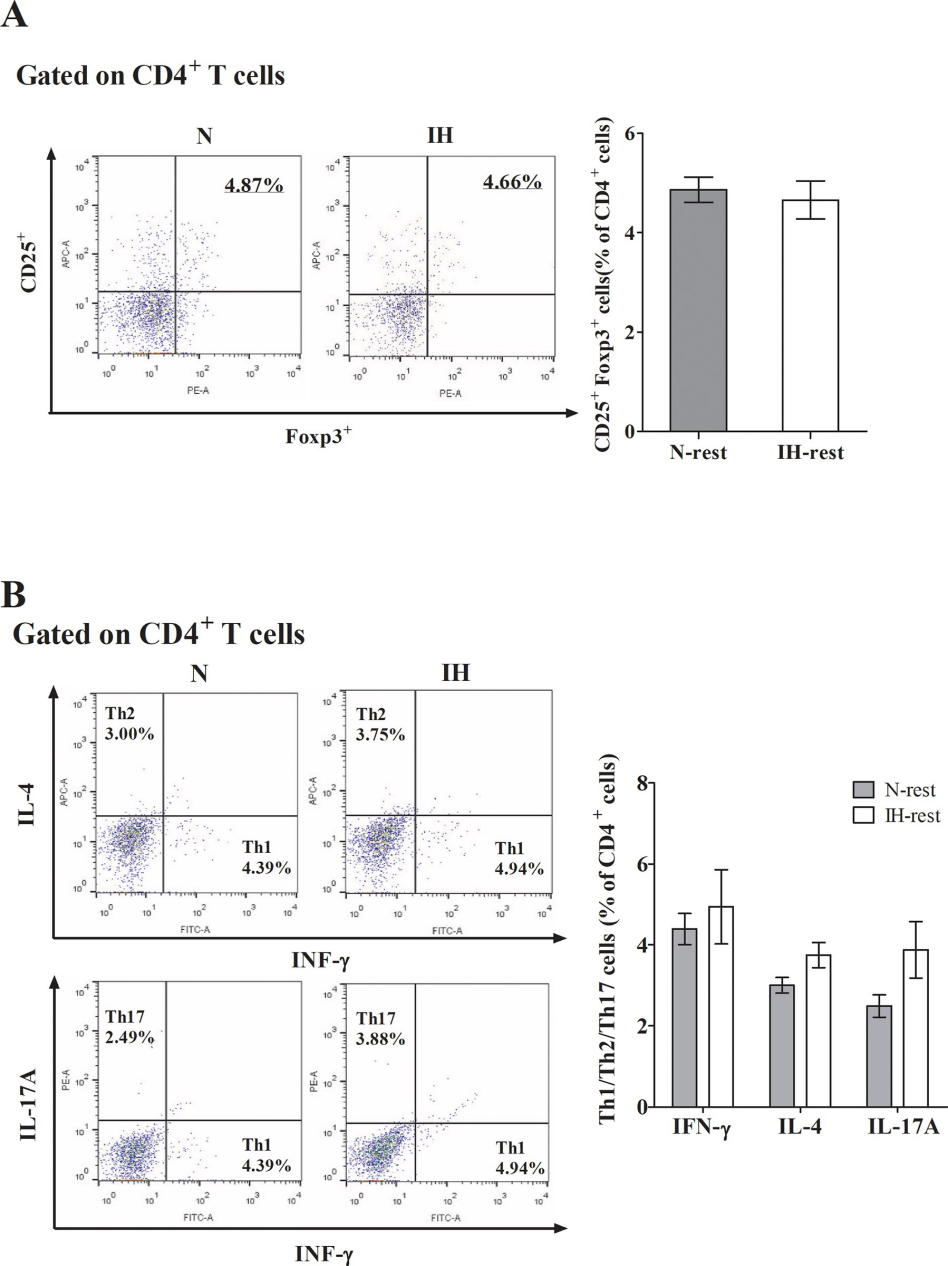
Frequency of regulatory T cells and Th1, Th2, and Th17 cytokine measured using flow cytometry in the OSA recovery mouse model. (A) Expression of CD4 ^+^ CD25 ^+^ Foxp3 ^+^ regulatory T cells did not significantly vary between the N-rest and IH-rest groups. (B) Expression of Th1, Th2, and Th17 cytokine did not significantly vary between the N-rest and IH-rest groups. Data are expressed as the mean ± S.E. (C57BL/6N mice, *n* = 6 per group). N, normoxia group. IH, intermittent hypoxia group.

To further strengthen the causality of the potential mechanism underlying T cell distribution changes induced by intermittent hypoxia (IH), we investigated the reversibility of this causal relationship by implementing a recovery period after cessation of IH stimulation. Following the four-week recovery period, we found no significant difference in the expression of CD4^+^, CD25^+^, and Foxp3^+^ Treg cells between the N-rest and IH-rest groups (Fig 6). The levels of IFN-γ, IL-4, and IL-17A were also not significantly different between the N-rest and IH-rest groups (Fig 6).

### Foxp3, NF-κB components, and inflammatory factors following hypoxia recovery

**Fig 7:**
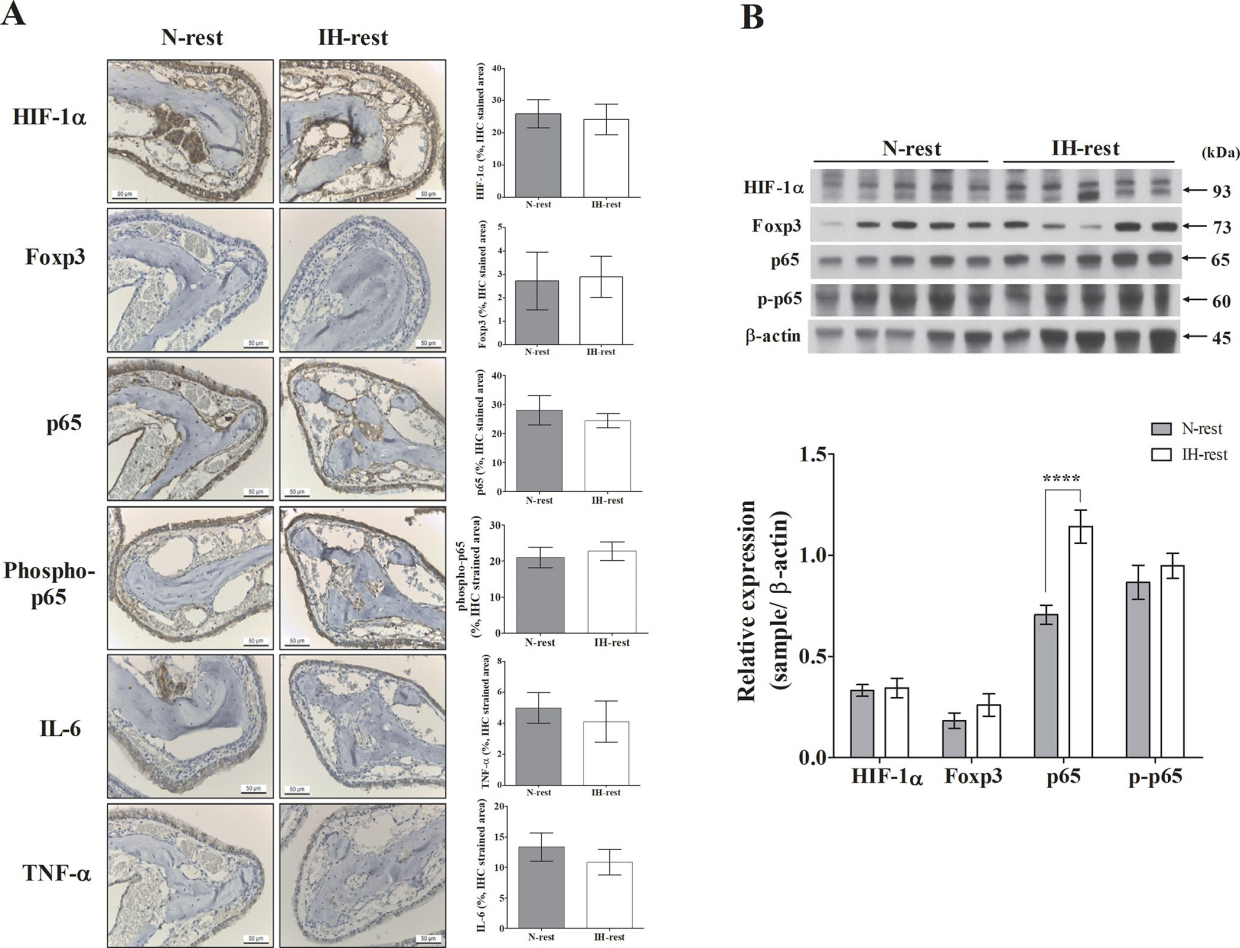
Histopathological analysis of nasal tissues and their quantification with western blotting in the OSA recovery mouse model. (A) Expression of HIF-1α, p65, phosphor-p65, IL-6 and TNF-α did not significantly vary between the N-rest and IH-rest groups. (B) Expression of HIF-1α, phospho-65, and p65 did not significantly vary between the N-rest and IH-rest groups. Data are expressed as the mean ± S.E. (C57BL/6N mice, *n* = 5 per group). N, normoxia group. IH, Intermittent hypoxia group. The significant difference between groups were indicated by asterisks (*p < 0.05, **p < 0.01, ***p < 0.001, ****p <0.0001).

Similarly, no significant difference in the expression of HIF-1α, hosphor-p65, and Foxp3 was observed between the N-rest and IH-rest groups. The level of p65 remained significantly higher in the IH group than in the N group; however, the extent of its increase was weakened (Fig 7). The levels of HIF-1α, Foxp3, molecules involved in the NF-κB pathway, and pro-inflammatory and inflammatory cytokines were not significantly different between the two groups.

## Discussion

We established an OSA mouse model using customized IH chambers to successfully mimic the mechanism of OSA in humans. In addition, the customized chamber has been designed to be large enough to contain mouse cages for experimental ease, reduction in gas consumption (thereby reducing the occurrence of possible hazards), ensuring a sufficient range of activity for the animals, and designing the system to be free of feed and water supply problems, thus fully complying with ethical guidelines for animal experimentation. The effect on sleep apnea was verified by changing the mouse Hb O_2_ saturation.

Our findings provided direct evidence of the effect of IH, the main pathomechanism of OSA, on the immune system. Overall, we found significantly increased Th17-related cytokine (IL-17) level and decreased Treg and transcription factor (Foxp3) levels in mice subjected to hypoxic conditions; however, there was no significant difference in the level of IFN-γ, Th 1 cell related cytokine. IL-4, a cytokine related to Th 2, was increased in the IH group, showing a predominant trend of Th 2 compared with Th 1. Tregs exert immunosuppressive functions by inhibiting excessive immune responses, while Th17 cells promote pro-inflammatory responses when warranted. Disruption of this delicate balance can precipitate immune dysregulation and contribute to the pathogenesis of various immune-mediated disorders. Thus, Tregs and Th17 cells interact in a complementary manner to sustain immune homeostasis. Our results suggest that IH, which is the primary mechanism underlying OSA, may exert a detrimental impact on immune homeostasis. We observed an increase in IFN-γ levels in the IH group compared to the normoxia group; however, the lack of statistical significance was attributed to the long-term stimulation of IH. This phenomenon may be explained by the role of IFN-γ primarily in the early stages of the inflammatory response.

It also showed an increase in HIF-1 expression and an increase in NF-κB pathway molecules. As reported, an elevation in HIF-1 molecules has been associated with increased IL-17 and decreased FoxP3 expression [26]. In our experiment, the observed increase in HIF-1 induced by intermittent hypoxia is also believed to contribute to an imbalance between Th17 and Treg cells. Furthermore, heightened HIF-1 expression is known to activate the NF-κB pathway [24]. We conducted an analysis of the activation of the NF-κB pathway through p65 and phospho-p65. Elevated levels of p65 and phospho-p65 suggest activation of the NF-κB pathway, which can be triggered by various stimuli, including pro-inflammatory cytokines, microbial pathogens, oxidative stress, and cellular damage. Upon activation, p65 translocates to the nucleus, where it regulates the transcription of target genes involved in inflammation, immune responses, and cell survival. Increased levels of p65 and phospho-p65 are commonly observed in medical conditions characterized by inflammation, autoimmune disorders, and cancer, wherein activation of the NF-κB pathway contributes to the expression of pro-inflammatory mediators and the recruitment of immune cells to sites of inflammation. Our experimental findings consistently demonstrated a trend towards increased levels of p65 and phospho-p65. Based on these results, we estimated that the elevation of HIF-1 activates the NF-κB pathway. Activation of the NF-κB pathway is closely linked to systemic inflammation, implying a potential mechanism through which intermittent hypoxia exacerbates inflammatory processes. These findings underscore the intricate interplay between hypoxia signaling pathways and immune dysregulation in the context of obstructive sleep apnea.

To strengthen the correlation between IH stimulation and the aforementioned results, we investigated the reversibility of these outcomes upon cessation of IH stimulation. After cessation of IH stimulation for a duration of 4 weeks, the imbalance between Th17 and Tregs resolved, and the state of Th2 dominance dissipated. Additionally, the levels of most inflammatory cytokines returned to measurements akin to those observed in the control group.

To the best of our knowledge, this is the first study to investigate the changes in Th17 and Treg cells during intermittent hypoxia in an OSA murine model. Previous studies have reported that in pediatric patients with OSA, Treg cells decrease and Th17 cells increase according to OSA severity [14, 27]. As one of the mechanisms regulating the expression of Tregs, which affect various immune system-related diseases, a mechanism by which Treg cells and Th17 cells are regulated like a seesaw by hypoxia has been reported [26]. Similarly, in intermittent hypoxia, where normoxia and hypoxia alternatively occur affecting simultaneously hypoxic and ROS mechanisms, increased levels of hypoxic molecules, such as HIF-1α, and inflammatory molecules were observed. It is thought to be controlled by the balance between Th17 cells and regulatory T cells. Intermittent hypoxia has been reported to cause increased levels of HIF molecules under hypoxic conditions, thus leading to inflammation via the activation of ROS and the NF-κB pathway [28–30]. In this study, we similarly observed increased levels of molecules related to the NF-κB pathway [31, 32].

Partial sleep restriction, including OSA, affects the regulation of signaling pathways associated with the immune system, and some of these changes appear to be long-lasting and may also contribute to inflammation-related pathologies [33]. Additionally, studies have shown that adult OSA can trigger autoimmune symptoms [11]. However, our findings suggest that the Th17/Treg imbalance can be resolved when hypoxic stimulation was ceased (i.e., recovery period). In this manner, higher Th17/Treg ratio can lead to the loss of tolerance and control, and ultimately to a persistent, low-level systemic inflammatory response characterized by autoimmune or allergic disorders.

This distorted T cell balance can explain diseases, such as allergies. Recently, the importance of the Th17/Treg balance in various autoimmune and inflammatory diseases has been identified [12, 34]. Clinically, cases of allergic rhinitis, which is affected by Th17 and Tregs, in patients with OSA is high in allergic diseases [9, 27, 35]. Until now, allergic rhinitis has been predominantly associated with upper airway symptoms and airway narrowing, potentially influencing the development or severity of OSA. However, our findings suggest that IH resulting from OSA may disrupt the balance between Th17 cells and Tregs, consequently exacerbating the symptoms of allergic rhinitis. Further investigation is warranted to delve deeper into this possibility and elucidate its underlying mechanisms.

Our study has some limitations. First, our proposed OSA mouse model was unable to different between sleep and non-sleep based on the mouse EEG measurement. There may be a difference from the actual sleep apnea because IH conditions were given during the normal routine sleep period of the mouse, not the actual sleep time. Second, a more definitive mechanism could be uncovered with the use of genetically engineered mice or the support of *in vitro* experiments. As a follow-up study, we are developing an appropriate *in vitro* cell culture chamber to validate our findings here. Third, we did not find any difference in the Th17/Treg balance and levels of related cytokines according to the degree or duration of application of IH. Such changes will more clearly show the effect of intermittent hypoxia on immune cell populations and can be induced by dramatically adjusting the IH severity.

## Conclusion

Our findings confirmed an increase in the Th17/Treg ratio following intermittent hypoxia, indicating that OSA may impact immune cell distribution and, consequently, related immunologic diseases. Furthermore, we observed that the immunologic imbalance can be significantly reversed upon cessation of IH stimulation. Therefore, in clinical practice, appropriate treatment of OSA to mitigate IH stimulation is crucial for preventing immunologic imbalance.

## Supporting information

S1 Raw images Original blot of protein expression of HIF-1α, p65, p-p65, Foxp3, and β-actin. (PDF)

S1 Table Saturation data for Fig 3. (PDF)
