# Supplementary material for: Intermittent hypoxia induces Th17/Treg imbalance in a murine model of obstructive sleep apnea
Source: PLoS One. 2024 Jun 24;19(6):e0305230. doi: 10.1371/journal.pone.0305230 (PMC11195984; doi:10.1371/journal.pone.0305230)

Fig. 5

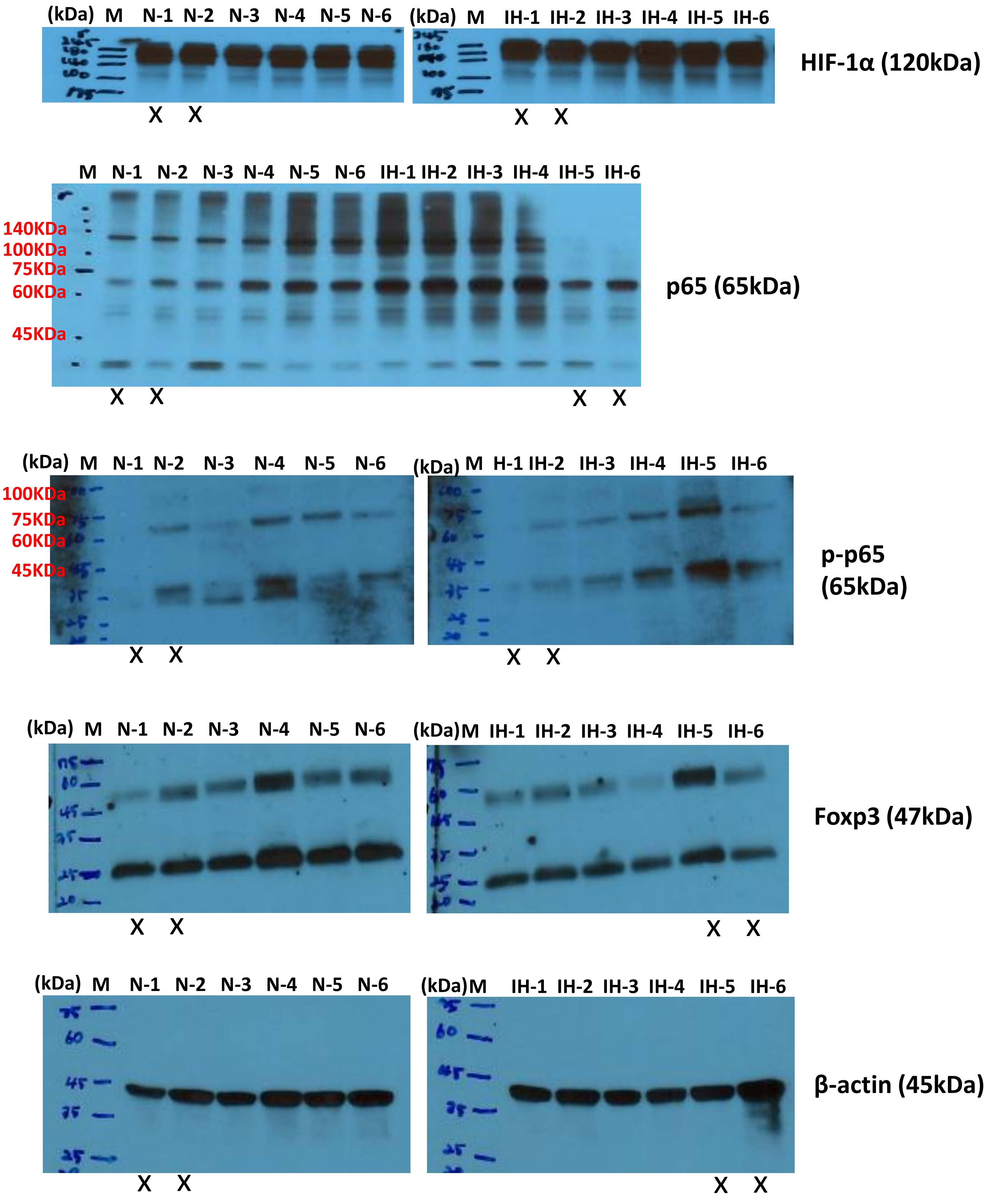

(kDa) M N-1 N-2 N-3 N-4 N-5 N-6
(kDa) M IH-1 IH-2 IH-3 IH-4 IH-5 IH-6
HIF-1α (120kDa)
X X
X X
M N-1 N-2 N-3 N-4 N-5 N-6 IH-1 IH-2 IH-3 IH-4 IH-5 IH-6
140KDa
100KDa
75KDa
60KDa
45KDa
p65 (65kDa)
X X
X X
(kDa) M N-1 N-2 N-3 N-4 N-5 N-6
(kDa) M H-1 IH-2 IH-3 IH-4 IH-5 IH-6
100KDa
75KDa
60KDa
45KDa
p-p65 (65kDa)
X X
X X
(kDa) M N-1 N-2 N-3 N-4 N-5 N-6
(kDa)M IH-1 IH-2 IH-3 IH-4 IH-5 IH-6
Foxp3 (47kDa)
X X
X X
(kDa) M N-1 N-2 N-3 N-4 N-5 N-6
(kDa)M IH-1 IH-2 IH-3 IH-4 IH-5 IH-6
β-actin (45kDa)
X X
X X

Fig. 5

HIF-1α (120kDa)

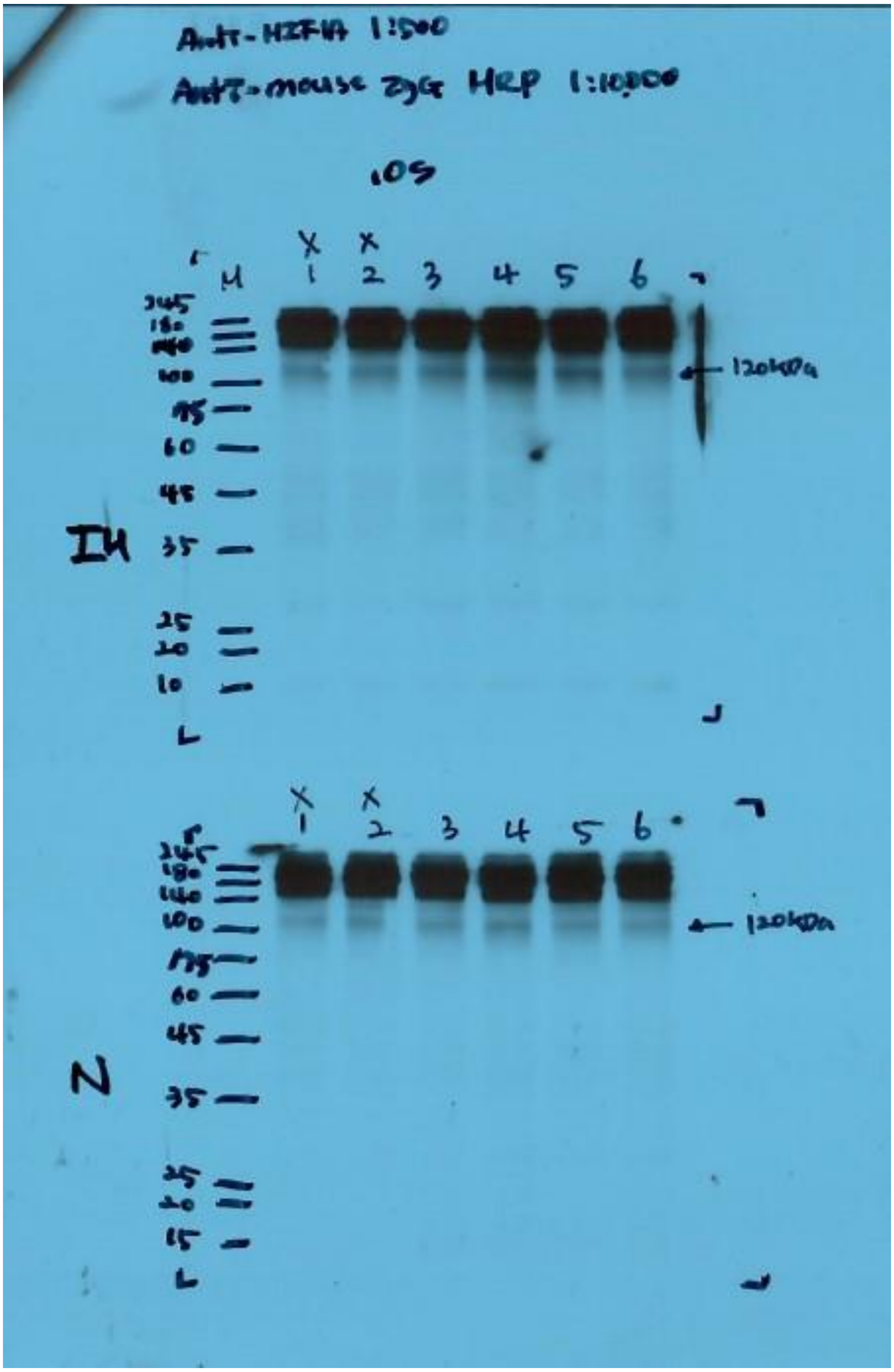

Fig. 5

Foxp3 (47kDa)

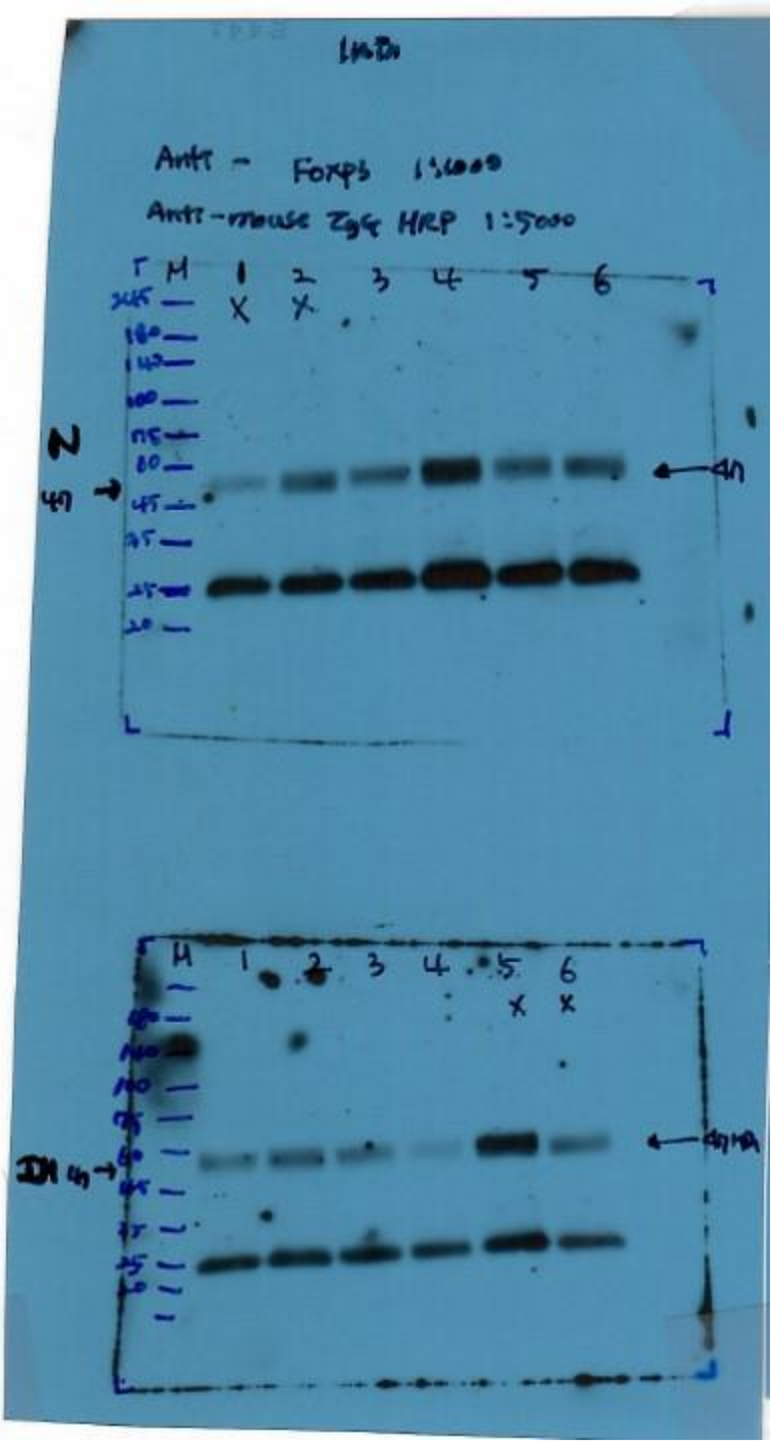

Fig. 5

p-p65
(65kDa)

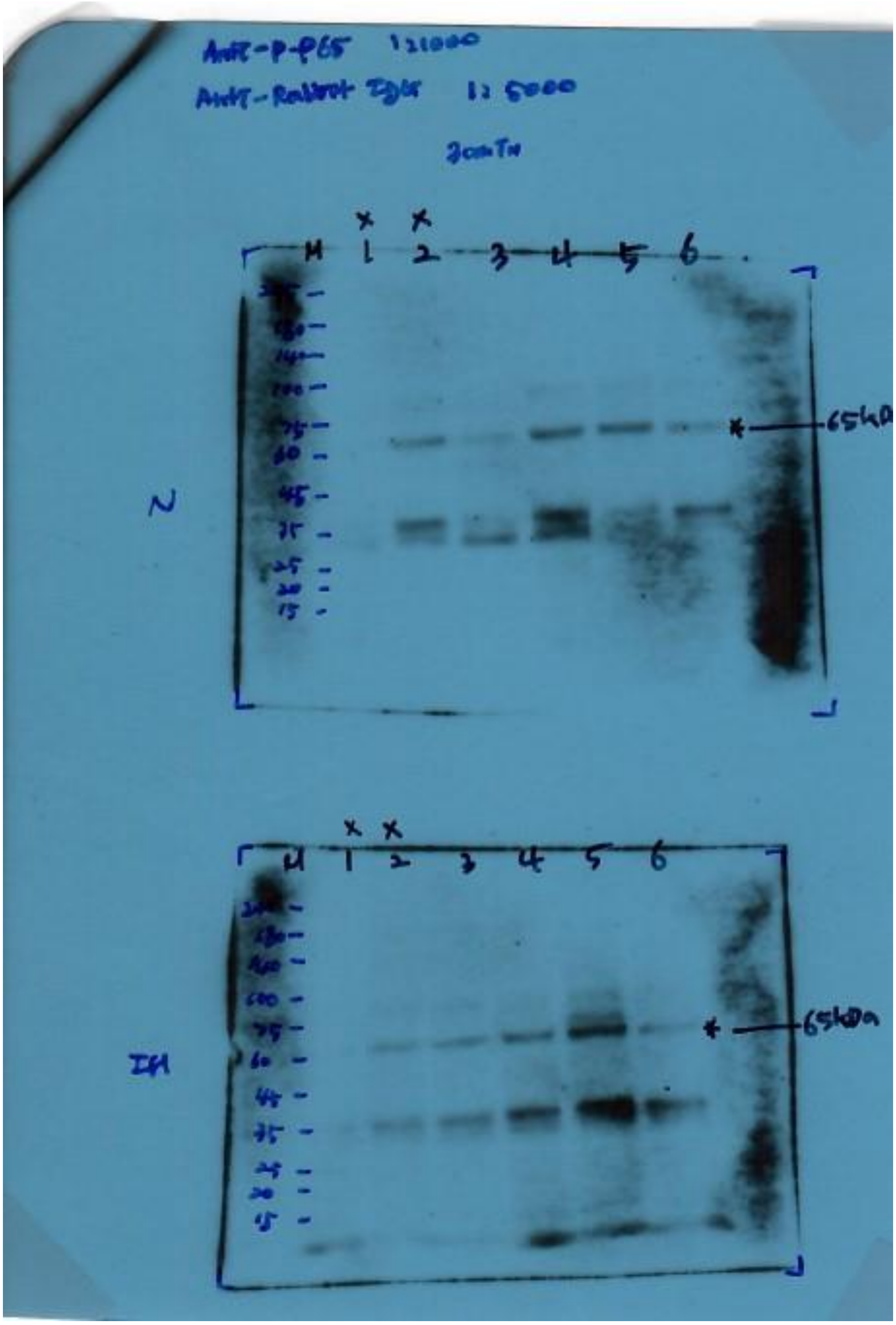

Fig. 5

p65 (65kDa)

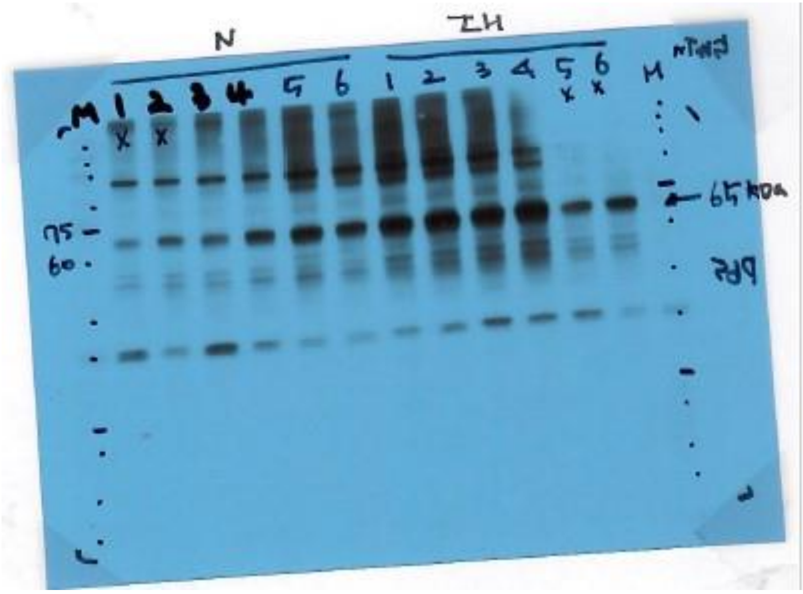

Fig. 5

β-actin (45kDa)

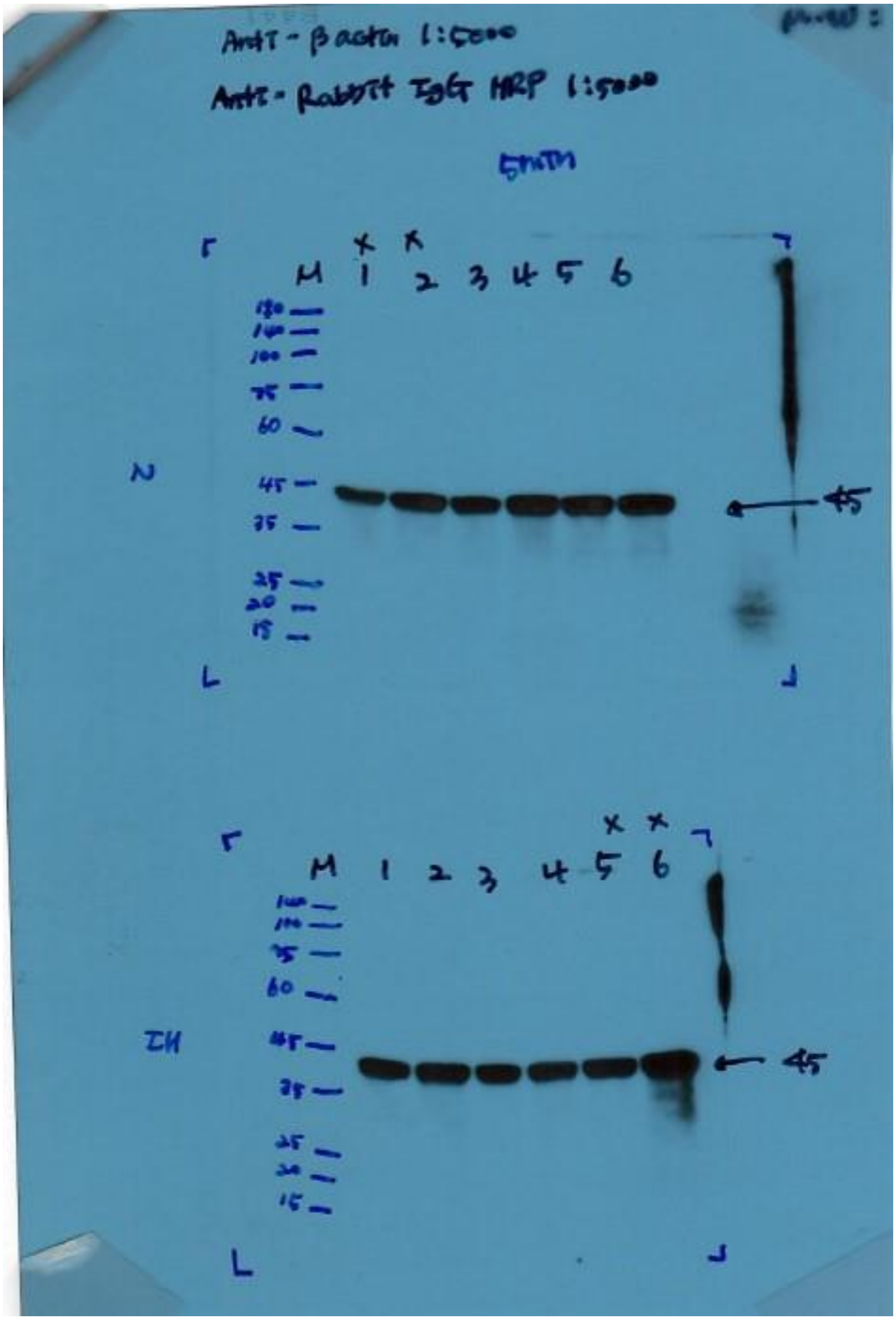

Fig. 7

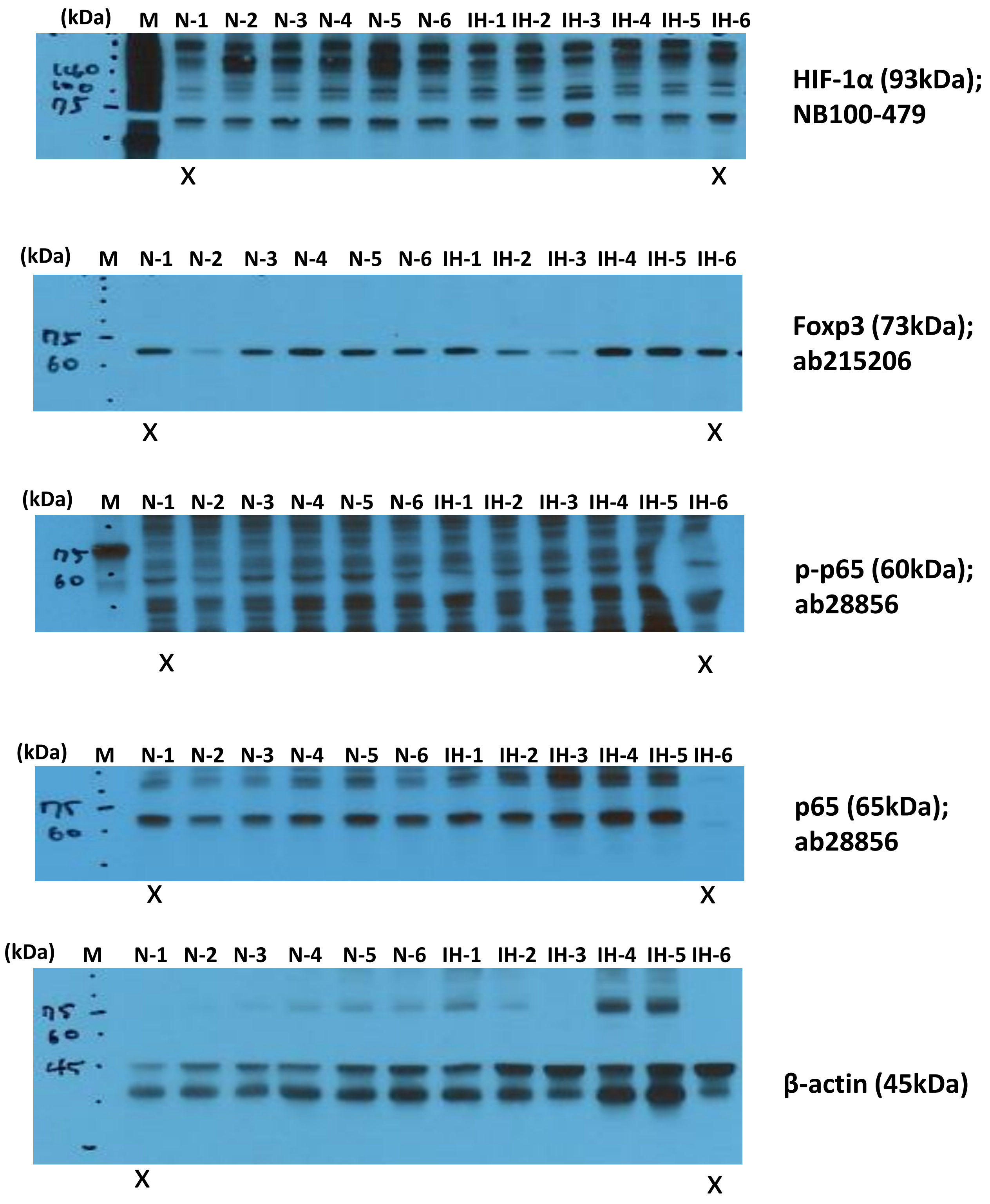
(kDa)
M N-1 N-2 N-3 N-4 N-5 N-6 IH-1 IH-2 IH-3 IH-4 IH-5 IH-6
140
100
75
X
X
HIF-1α (93kDa);
NB100-479
(kDa)
M N-1 N-2 N-3 N-4 N-5 N-6 IH-1 IH-2 IH-3 IH-4 IH-5 IH-6
75
60
X
X
Foxp3 (73kDa);
ab215206
(kDa)
M N-1 N-2 N-3 N-4 N-5 N-6 IH-1 IH-2 IH-3 IH-4 IH-5 IH-6
75
60
X
X
p-p65 (60kDa);
ab28856
(kDa)
M N-1 N-2 N-3 N-4 N-5 N-6 IH-1 IH-2 IH-3 IH-4 IH-5 IH-6
75
60
X
X
p65 (65kDa);
ab28856
(kDa)
M N-1 N-2 N-3 N-4 N-5 N-6 IH-1 IH-2 IH-3 IH-4 IH-5 IH-6
75
60
45
X
X
β-actin (45kDa)

Fig. 7

HIF-1α (93kDa);
NB100-479

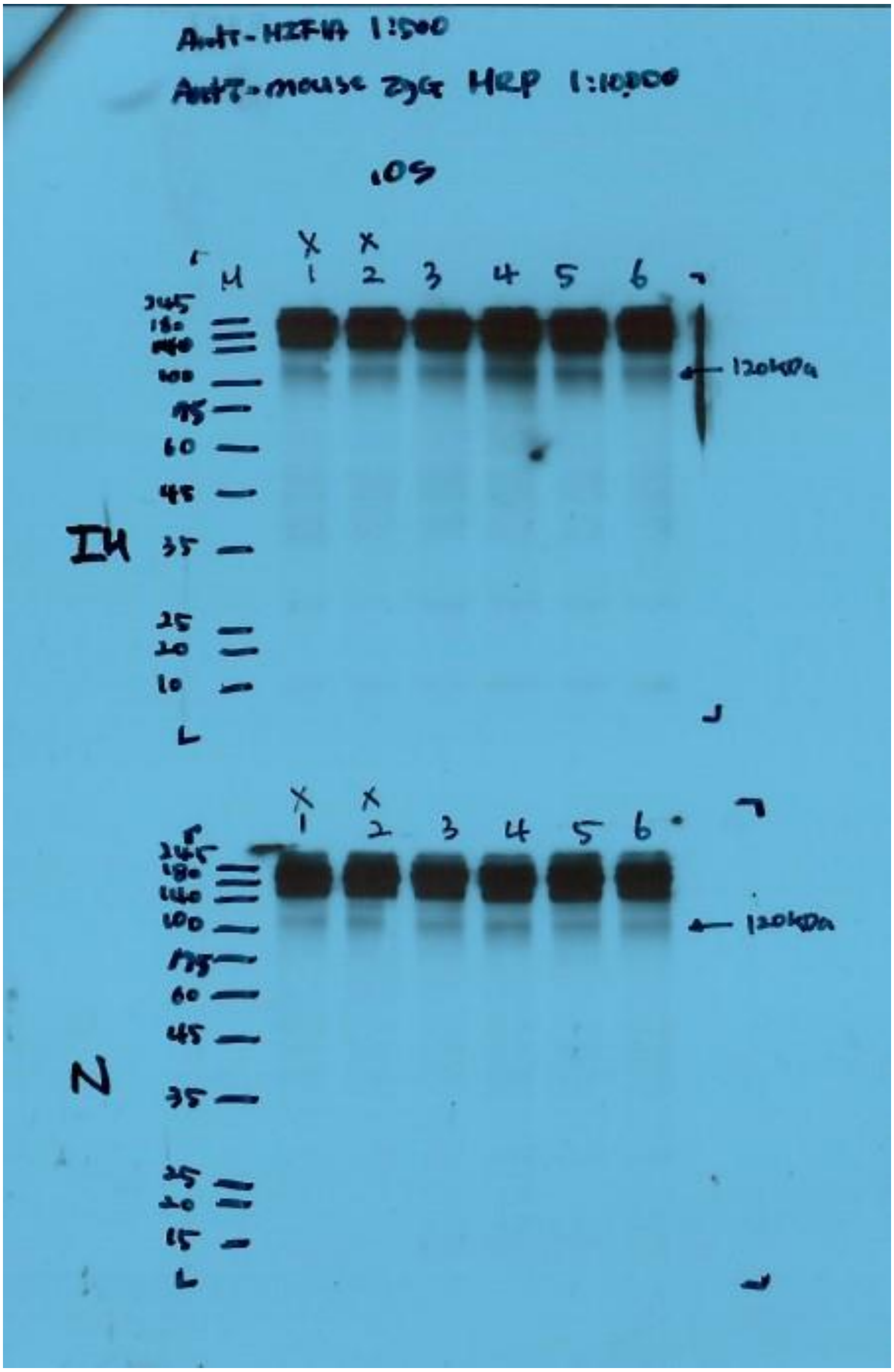

Fig. 7

Foxp3 (73kDa);
ab215206

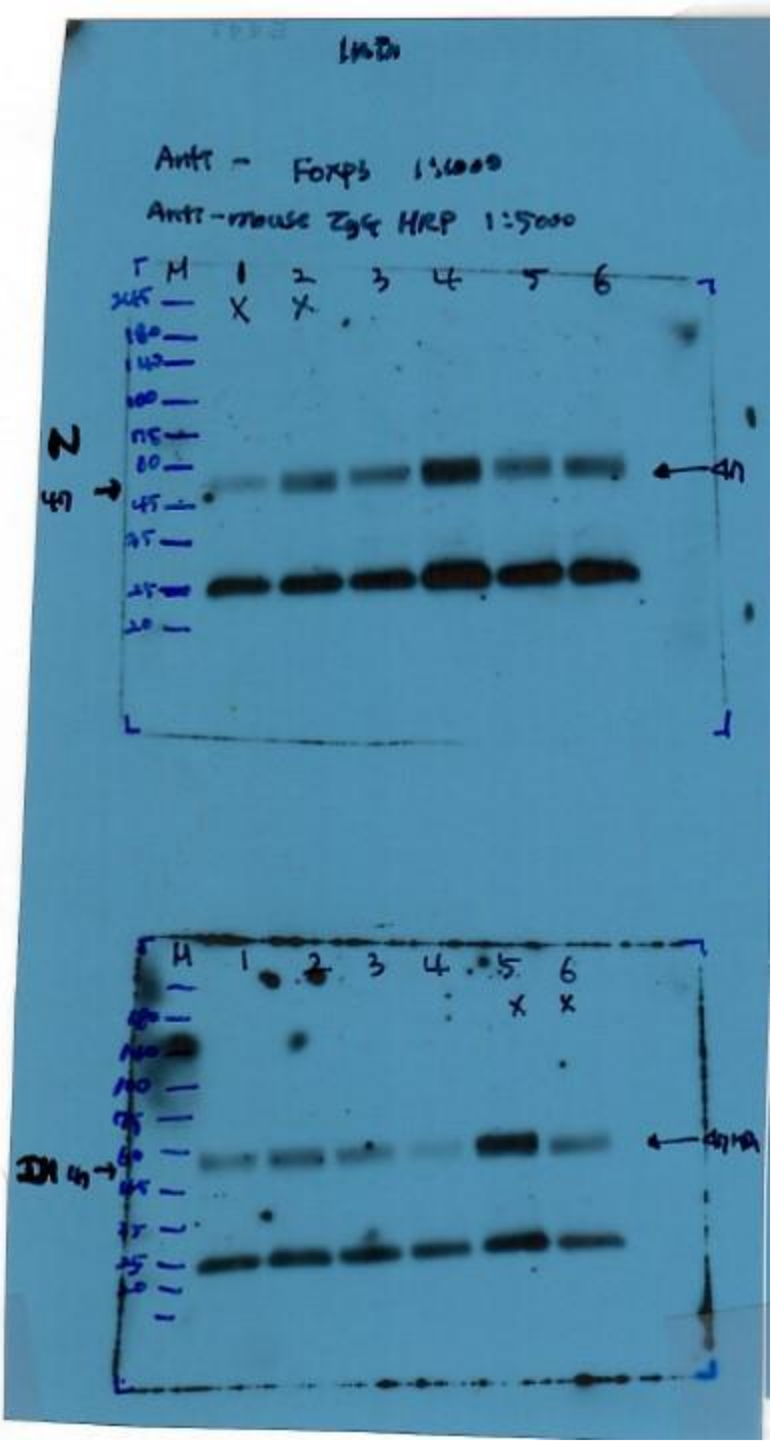

Fig. 7

p-p65 (60kDa);
ab28856

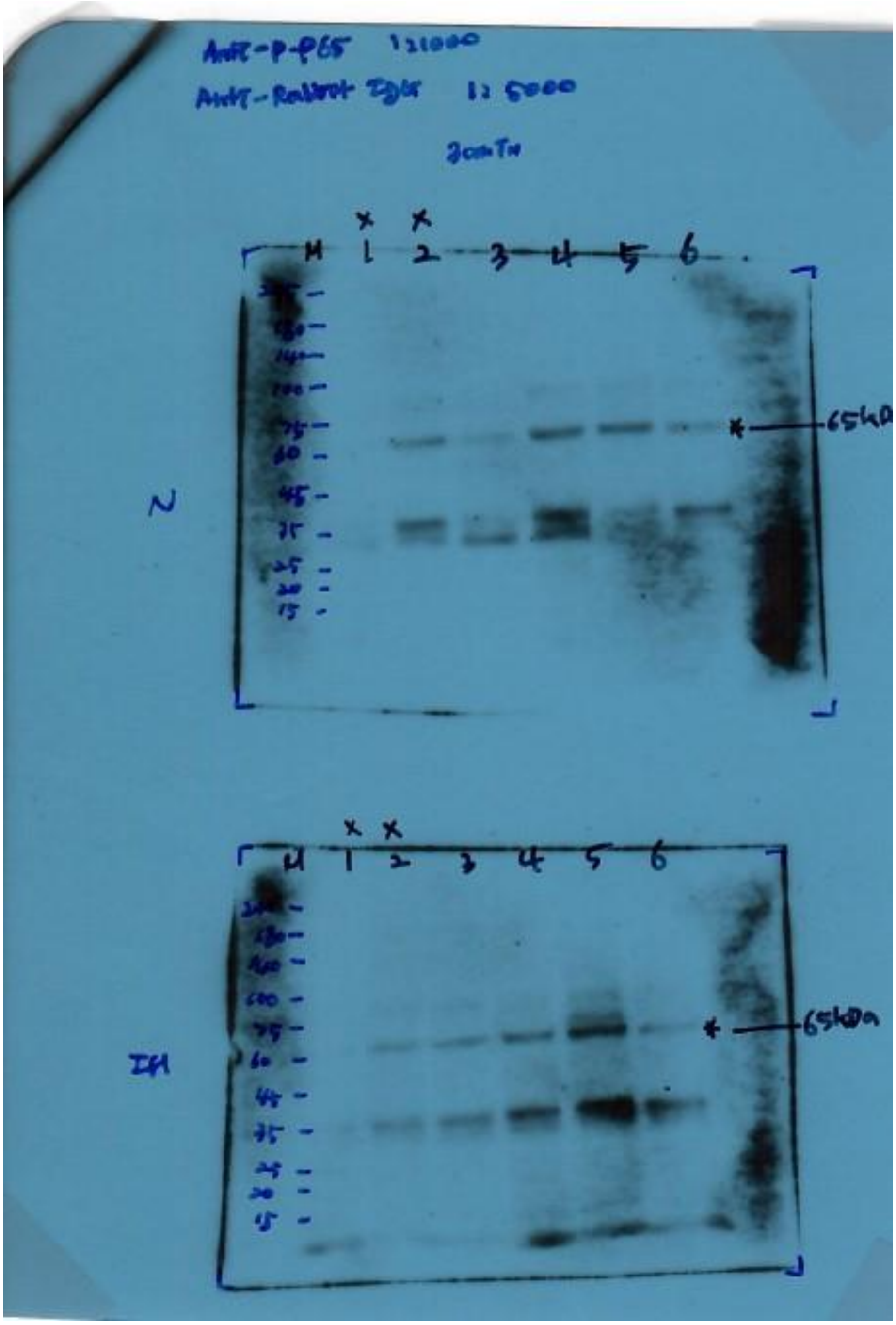

Fig. 7

p65 (65kDa);
ab28856

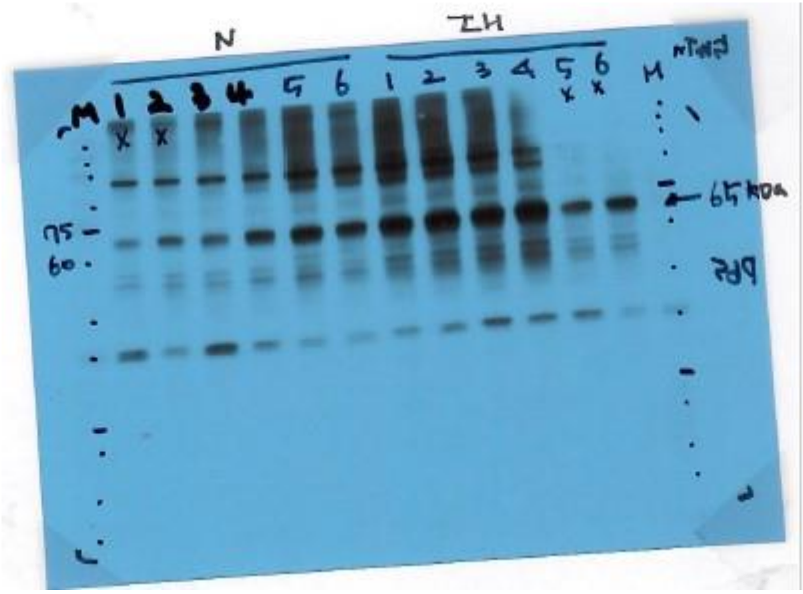

Fig. 7

β-actin (45kDa)

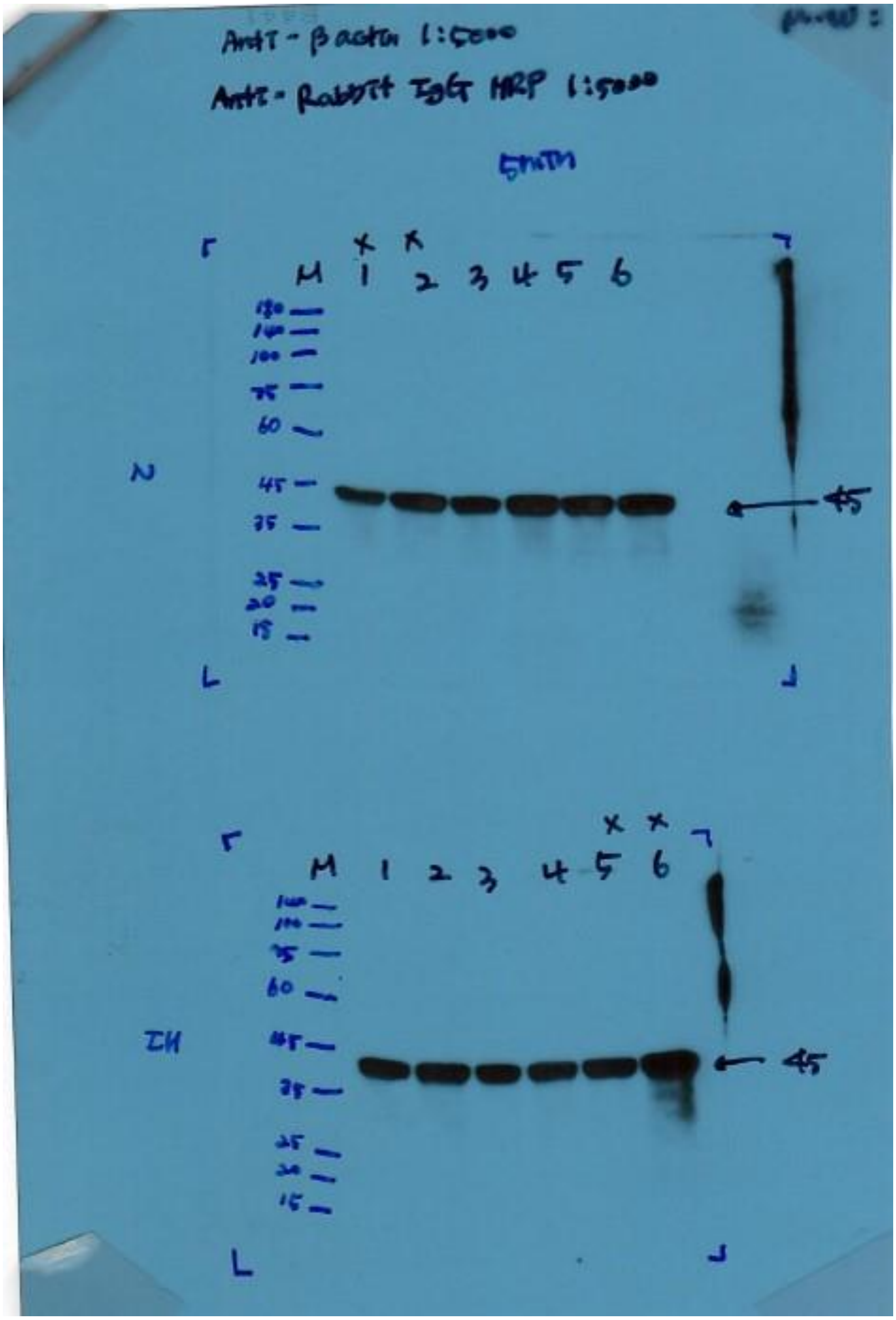

Supplement: S1 Raw images — (PDF) [file pone.0305230.s001.pdf]
