# Supplementary material for: Intermittent hypoxia induces Th17/Treg imbalance in a murine model of obstructive sleep apnea
Source: PLoS One. 2024 Jun 24;19(6):e0305230. doi: 10.1371/journal.pone.0305230 (PMC11195984; doi:10.1371/journal.pone.0305230)
Supplement: S1 Table — (PDF) [file pone.0305230.s002.pdf]

		N									IH						
O2	SpO2(%)	SpO2	SpO2	SpO2	SpO2	AVE	SD	SE	O2	SpO2(%)	SpO2	SpO2	SpO2	SpO2	AVE	SD	SE
21	99	98	99	99	98	98.6	0.547723	0.244949	20.3	95	99	99	97	94	96.8	2.28035	1.0198
21	96	92	90	98	97	94.6	3.435113	1.536229	20.3	96	98	98	92	91	95	3.31662	1.48324
21	95	93	92	97	97	94.8	2.280351	1.019804	20.3	91	98	95	89	92	93	3.53553	1.58114
21	99	96	93	95	93	95.2	2.48998	1.113553	20.3	90	96	94	97	94	94.2	2.68328	1.2
21	92	96	93	96	97	94.8	2.167948	0.969536	20.2	86	97	94	89	87	90.6	4.72229	2.11187
21	96	94	96	95	96	95.4	0.894427	0.4	18.9	85	95	93	87	86	89.2	4.49444	2.00998
21	91	92	96	95	92	93.2	2.167948	0.969536	16	79	96	92	81	85	86.6	7.23187	3.23419
21	88	96	96	97	94	94.2	3.63318	1.624808	13.2	74	94	91	71	79	81.8	10.2323	4.57602
21	95	97	97	96	89	94.8	3.34664	1.496663	10.8	77	84	89	66	72	77.6	9.1815	4.10609
21	99	93	96	93	90	94.2	3.420526	1.529706	8.7	73	79	86	65	62	73	9.87421	4.41588
21	95	90	96	92	92	93	2.44949	1.095445	7.3	65	83	83	64	60	71	11.1131	4.96991
21	96	99	98	91	94	95.6	3.209361	1.43527	5.9	60	85	86	68	65	72.8	11.9457	5.34228
21	94	94	96	90	96	94	2.44949	1.095445	5	61	82	82	70	73	73.6	8.84873	3.95727
21	99	92	95	91	99	95.2	3.768289	1.68523	4.7	60	80	80	66	68	70.8	8.89944	3.97995
21	99	95	94	92	98	95.6	2.880972	1.28841	4.7	60	66	73	68	67	66.8	4.65833	2.08327
21	87	92	94	95	94	92.4	3.209361	1.43527	4.7	60	60	60	60	61	60.2	0.44721	0.2
21	99	99	96	92	93	95.8	3.271085	1.462874	4.7	61	60	62	58	64	61	2.23607	1
21	94	93	95	91	92	93	1.581139	0.707107	6.7	68	60	65	81	71	69	7.84219	3.50714
21	90	91	96	98	95	94	3.391165	1.516575	10.9	77	66	70	87	80	76	8.27647	3.70135
21	91	94	96	90	91	92.4	2.50998	1.122497	14	80	73	88	86	81	81.6	5.85662	2.61916
21	97	96	92	93	92	94	2.345208	1.048809	16.2	89	76	87	94	67	82.6	10.9225	4.88467
21	93	97	95	91	93	93.8	2.280351	1.019804	17.4	97	75	88	87	65	82.4	12.482	5.58211
21	89	94	90	97	95	93	3.391165	1.516575	18.4	96	76	87	88	64	82.2	12.4177	5.55338
21	99	95	95	94	90	94.6	3.209361	1.43527	19	97	78	89	94	69	85.4	11.6748	5.22111
21	97	97	99	96	92	96.2	2.588436	1.157584	19.4	96	77	91	95	73	86.4	10.6677	4.77074
21	92	98	99	99	91	95.8	3.962323	1.772005	19.6	97	79	90	96	77	87.8	9.36483	4.18808
21	97	97	93	93	89	93.8	3.34664	1.496663	19.8	96	81	88	97	81	88.6	7.76531	3.47275
21	94	95	90	95	99	94.6	3.209361	1.43527	20	98	80	88	97	88	90.2	7.42967	3.32265
21	98	96	95	99	92	96	2.738613	1.224745	20	95	79	90	91	87	88.4	5.98331	2.67582
21	97	89	93	98	93	94	3.605551	1.612452	19.1	89	78	89	87	83	85.2	4.71169	2.10713
21	95	95	93	94	92	93.8	1.30384	0.583095	16.1	82	76	88	88	80	82.8	5.21536	2.33238
21	97	92	99	95	95	95.6	2.607681	1.16619	13.2	82	80	87	87	66	80.4	8.61974	3.85487
21	96	96	98	94	93	95.4	1.949359	0.87178	11	74	76	76	90	70	77.2	7.56307	3.38231
21	91	94	97	96	94	94.4	2.302173	1.029563	9	70	72	75	82	79	75.6	4.9295	2.20454
21	99	96	93	94	93	95	2.54951	1.140175	7.3	69	67	70	79	66	70.2	5.1672	2.31084
21	94	97	92	92	94	93.8	2.04939	0.916515	5.9	68	65	68	74	60	67	5.09902	2.28035
21	95	95	93	98	98	95.8	2.167948	0.969536	4.9	60	62	60	68	68	63.6	4.09878	1.83303
21	93	94	99	93	95	94.8	2.48998	1.113553	4.5	60	60	60	67	67	62.8	3.83406	1.71464
21	96	93	94	98	96	95.4	1.949359	0.87178	4.7	69	61	65	67	62	64.8	3.34664	1.49666
21	94	94	95	93	97	94.6	1.516575	0.678233	6.2	73	64	95	68	90	78	13.7295	6.14003

21	93	93	97	99	98	96	2.828427	1.264911	9.9	82	65	70	72	95	76.8	11.9038	5.32353
21	94	96	96	98	95	95.8	1.48324	0.663325	13.7	86	66	71	79	94	79.2	11.2561	5.03389
21	98	93	94	98	98	96.2	2.48998	1.113553	16.1	88	66	73	85	88	80	9.97497	4.46094
21	99	94	99	91	92	95	3.807887	1.702939	17.4	95	70	74	86	90	83	10.6301	4.75395
21	98	96	99	90	97	96	3.535534	1.581139	18.5	98	74	77	88	88	85	9.64365	4.31277
21	99	94	99	91	97	96	3.464102	1.549193	19.1	99	78	80	89	90	87.2	8.46759	3.78682
21	99	94	99	89	92	94.6	4.393177	1.964688	19.5	99	88	83	92	91	90.6	5.85662	2.61916
21	99	96	94	97	97	96.6	1.81659	0.812404	19.6	99	94	88	95	92	93.6	4.03733	1.80555
21	99	93	94	93	91	94	3	1.341641	19.8	99	95	90	99	93	95.2	3.89872	1.74356
21	99	96	92	93	92	94.4	3.04959	1.363818	19.9	98	96	95	94	94	95.4	1.67332	0.74833
21	94	91	96	98	94	94.6	2.607681	1.16619	20	97	95	94	87	93	93.2	3.76829	1.68523
21	99	97	99	90	93	95.6	3.974921	1.777639	19	95	86	90	83	88	88.4	4.50555	2.01494
21	90	95	97	91	95	93.6	2.966479	1.32665	15.9	94	81	88	81	82	85.2	5.71839	2.55734
21	92	96	97	95	98	95.6	2.302173	1.029563	13.2	88	80	86	78	60	78.4	11.0815	4.9558
21	94	91	95	95	93	93.6	1.67332	0.748331	10.9	84	79	84	65	75	77.4	7.89303	3.52987
21	91	94	90	96	97	93.6	3.04959	1.363818	9	82	77	80	61	72	74.4	8.38451	3.74967
21	99	97	92	98	94	96	2.915476	1.30384	7.3	78	76	76	75	73	75.6	1.81659	0.8124
21	93	98	90	99	98	95.6	3.911521	1.749286	5.9	80	75	70	65	61	70.2	7.59605	3.39706
21	95	95	97	99	99	97	2	0.894427	4.8	66	76	68	62	58	66	6.78233	3.03315
21	96	94	99	99	96	96.8	2.167948	0.969536	4.7	60	75	65	60	62	64.4	6.26897	2.80357
21	95	95	98	99	97	96.8	1.788854	0.8	4.5	60	76	60	60	60	63.2	7.15542	3.2
21	98	94	96	99	95	96.4	2.073644	0.927362	4.7	60	74	60	65	61	64	5.95819	2.66458
21	99	92	97	98	90	95.2	3.962323	1.772005	4.7	60	88	66	60	60	66.8	12.1326	5.42586
21	98	94	90	90	93	93	3.316625	1.48324	6.1	69	83	70	69	69	72	6.16441	2.75681
21	99	96	95	98	92	96	2.738613	1.224745	10.3	71	79	71	68	68	71.4	4.50555	2.01494
21	99	95	92	95	95	95.2	2.48998	1.113553	13.6	72	84	72	72	68	73.6	6.0663	2.71293
21	94	92	92	92	96	93.2	1.788854	0.8	15.8	76	84	76	82	68	77.2	6.26099	2.8
21	96	94	90	91	98	93.8	3.34664	1.496663	17.3	81	86	82	87	69	81	7.17635	3.20936
21	97	94	92	99	97	95.8	2.774887	1.240967	18.4	84	87	86	88	68	82.6	8.29458	3.70945
21	98	97	94	98	96	96.6	1.67332	0.748331	19	86	87	88	90	79	86	4.1833	1.87083
21	98	96	93	96	96	95.8	1.788854	0.8	19.5	92	88	95	96	86	91.4	4.3359	1.93907
21	98	97	99	89	93	95.2	4.147288	1.854724	19.6	99	89	99	97	89	94.6	5.17687	2.31517
21	96	94	95	98	98	96.2	1.788854	0.8	19.8	99	90	99	92	91	94.2	4.43847	1.98494
21	97	93	98	93	96	95.4	2.302173	1.029563	20	98	91	99	87	92	93.4	5.02991	2.24944
21	96	91	90	96	97	94	3.24037	1.449138	19.2	94	91	92	75	89	88.2	7.59605	3.39706
21	97	96	98	97	99	97.4	1.140175	0.509902	16.1	92	89	91	78	78	85.6	7.0214	3.14006
21	90	97	94	95	92	93.6	2.701851	1.208305	13.4	89	88	89	77	73	83.2	7.62889	3.41174
21	99	98	97	96	96	97.2	1.30384	0.583095	11.1	88	87	88	72	68	80.6	9.78775	4.37721
21	99	94	88	97	93	94.2	4.207137	1.881489	9	79	89	87	67	64	77.2	11.3666	5.08331
21	99	96	91	98	94	95.6	3.209361	1.43527	7.6	75	90	89	73	61	77.6	12.1161	5.41849
21	92	97	97	98	93	95.4	2.701851	1.208305	6.2	73	89	90	68	62	76.4	12.5817	5.62672
21	97	93	98	98	92	95.6	2.880972	1.28841	5	73	89	89	89	60	80	13.1529	5.88218

21	99	95	96	95	89	94.8	3.63318	1.624808	4.5	74	88	89	71	60	76.4	12.2188	5.46443
21	98	96	94	99	92	95.8	2.863564	1.280625	4.5	69	82	88	69	60	73.6	11.2383	5.02593
21	99	97	99	98	98	98.2	0.83666	0.374166	4.7	68	78	71	61	61	67.8	7.19027	3.21559
21	98	93	93	92	96	94.4	2.50998	1.122497	4.7	61	71	71	60	60	64.6	5.85662	2.61916
21	98	97	99	96	93	96.6	2.302173	1.029563	5.7	61	71	73	61	65	66.2	5.5857	2.498
21	89	98	98	94	97	95.2	3.834058	1.714643	10.1	60	73	74	67	86	72	9.61769	4.30116
21	94	89	95	97	96	94.2	3.114482	1.392839	13.5	60	74	77	78	88	75.4	10.0896	4.51221
21	99	90	95	99	91	94.8	4.266146	1.907878	15.7	69	77	79	79	94	79.6	9.04434	4.04475
21	99	98	99	99	98	98.6	0.547723	0.244949	17.3	69	79	78	80	99	81	10.9772	4.90918
21	99	96	92	98	95	96	2.738613	1.224745	18.3	72	78	81	80	99	82	10.1242	4.52769
21	99	95	98	97	89	95.6	3.974921	1.777639	19	84	81	83	79	97	84.8	7.0852	3.1686
21	96	94	93	97	91	94.2	2.387467	1.067708	19.6	91	83	84	83	96	87.4	5.85662	2.61916
21	99	96	91	94	90	94	3.674235	1.643168	20	94	84	87	93	95	90.6	4.82701	2.1587
21	95	96	98	95	96	96	1.224745	0.547723	20.2	95	84	88	96	90	90.6	4.97996	2.22711
21	90	97	92	90	95	92.8	3.114482	1.392839	20.2	98	87	90	97	92	92.8	4.65833	2.08327
21	98	95	97	99	94	96.6	2.073644	0.927362	20.2	99	88	95	87	90	91.8	5.06952	2.26716
21	97	94	91	97	90	93.8	3.271085	1.462874	19.6	90	86	86	81	94	87.4	4.87852	2.18174
21	99	96	96	95	97	96.6	1.516575	0.678233	16.7	95	86	86	87	85	87.8	4.08656	1.82757
21	96	94	93	98	99	96	2.54951	1.140175	13.9	94	86	87	84	81	86.4	4.82701	2.1587
21	96	97	96	98	94	96.2	1.48324	0.663325	11.2	92	87	87	76	74	83.2	7.79102	3.48425
21	99	92	97	92	98	95.6	3.361547	1.50333	9.3	88	87	86	81	72	82.8	6.6106	2.95635
21	99	97	99	96	92	96.6	2.880972	1.28841	7.6	86	86	87	76	73	81.6	6.58027	2.94279
21	99	98	96	95	97	97	1.581139	0.707107	6.1	75	87	88	72	74	79.2	7.66159	3.42637
21	93	95	96	95	99	95.6	2.19089	0.979796	5.1	67	88	87	72	61	75	12.0623	5.39444
21	96	96	96	97	99	96.8	1.30384	0.583095	4.7	64	87	60	60	60	66.2	11.7558	5.25738
21	96	98	97	94	97	96.4	1.516575	0.678233	4.5	60	60	66	60	60	61.2	2.68328	1.2
21	93	91	97	95	99	95	3.162278	1.414214	4.7	60	66	60	60	60	61.2	2.68328	1.2
21	91	92	95	96	99	94.6	3.209361	1.43527	5.5	62	60	66	70	62	64	4	1.78885
21	99	94	99	97	97	97.2	2.04939	0.916515	9.7	61	66	75	75	70	69.4	6.02495	2.69444
21	89	97	93	95	94	93.6	2.966479	1.32665	12.9	60	75	76	77	72	72	6.96419	3.11448
21	99	96	92	97	99	96.6	2.880972	1.28841	15.3	60	76	78	80	73	73.4	7.92465	3.54401
21	98	95	93	94	95	95	1.870829	0.83666	17	60	78	80	83	74	75	9	4.02492
21	92	97	97	96	96	95.6	2.073644	0.927362	18.3	88	86	87	84	75	84	5.24404	2.34521
21	99	96	92	92	99	95.6	3.507136	1.568439	19	88	87	89	82	75	84.2	5.80517	2.59615
21	93	95	99	97	91	95	3.162278	1.414214	19.4	90	89	92	92	77	88	6.2849	2.81069
21	95	97	92	89	99	94.4	3.974921	1.777639	19.6	91	88	95	94	78	89.2	6.83374	3.05614
21	98	94	97	99	94	96.4	2.302173	1.029563	19.8	92	89	99	92	89	92.2	4.08656	1.82757
21	97	97	90	98	98	96	3.391165	1.516575	20	99	90	99	88	90	93.2	5.35724	2.39583
21	98	96	99	96	93	96.4	2.302173	1.029563	20	99	92	99	98	91	95.8	3.96232	1.772
21	96	97	99	98	92	96.4	2.701851	1.208305	19.6	86	91	95	81	87	88	5.2915	2.36643
21	96	95	93	92	93	93.8	1.643168	0.734847	16.9	84	89	89	87	71	84	7.54983	3.37639
21	97	96	99	93	99	96.8	2.48998	1.113553	13.9	82	88	88	84	70	82.4	7.4027	3.31059

21	99	95	97	96	92	95.8	2.588436	1.157584	11.4	74	87	87	76	77	80.2	6.30079	2.8178
21	96	96	98	96	93	95.8	1.788854	0.8	9.4	69	89	85	81	67	78.2	9.75705	4.36348
21	95	95	99	96	93	95.6	2.19089	0.979796	7.7	67	90	76	76	65	74.8	9.88433	4.42041
21	91	96	99	98	94	95.6	3.209361	1.43527	6.3	64	89	70	72	62	71.4	10.6677	4.77074
21	95	98	99	97	98	97.4	1.516575	0.678233	5.1	63	89	60	72	60	68.8	12.3167	5.50818
21	97	97	99	98	97	97.6	0.894427	0.4	4.7	62	88	60	60	60	66	12.3288	5.51362
21	98	98	94	94	93	95.4	2.408319	1.077033	5.1	62	71	62	70	60	65	5.09902	2.28035
21	97	94	98	98	94	96.2	2.04939	0.916515	9	64	71	64	75	60	66.8	6.05805	2.70924
21	97	89	93	95	92	93.2	3.03315	1.356466	12.6	65	73	65	77	70	70	5.19615	2.32379
21	94	91	99	91	97	94.4	3.577709	1.6	15.4	65	74	65	80	69	70.6	6.42651	2.87402
21	95	96	99	94	93	95.4	2.302173	1.029563	17.1	65	77	65	83	77	73.4	8.04984	3.6
21	95	92	99	95	99	96	3	1.341641	18.2	74	79	74	84	83	78.8	4.76445	2.13073
21	90	96	99	96	92	94.6	3.577709	1.6	18.9	72	78	72	82	90	78.8	7.56307	3.38231
21	94	92	95	93	94	93.6	1.140175	0.509902	19.3	73	81	73	92	94	82.6	10.0648	4.50111
21	98	94	99	99	91	96.2	3.563706	1.593738	19.6	79	83	79	94	95	86	7.93725	3.54965
21	90	93	91	98	93	93	3.082207	1.378405	19.8	86	84	86	92	98	89.2	5.76194	2.57682
21	96	94	93	95	99	95.4	2.302173	1.029563	19.9	97	84	97	88	96	92.4	6.02495	2.69444
21	96	96	90	97	99	95.6	3.361547	1.50333	20	99	87	99	98	93	95.2	5.21536	2.33238
21	99	98	99	99	99	98.8	0.447214	0.2	19.8	94	88	94	81	94	90.2	5.76194	2.57682
21	98	96	98	91	98	96.2	3.03315	1.356466	17.3	93	86	93	87	92	90.2	3.42053	1.52971
21	94	95	95	97	98	95.8	1.643168	0.734847	14.3	90	86	90	84	88	87.6	2.60768	1.16619
21	89	95	89	98	98	93.8	4.549725	2.034699	11.8	86	86	86	76	86	84	4.47214	2
21	93	90	94	97	95	93.8	2.588436	1.157584	9.6	89	87	89	81	83	85.8	3.63318	1.62481
21	90	94	92	89	99	92.8	3.962323	1.772005	7.9	88	87	88	76	81	84	5.33854	2.38747
21	95	98	99	97	99	97.6	1.67332	0.748331	6.3	86	86	86	72	80	82	6.16441	2.75681
21	99	90	98	91	94	94.4	4.037326	1.805547	5.1	79	87	79	72	74	78.2	5.80517	2.59615
21	96	96	96	95	99	96.4	1.516575	0.678233	4.6	68	88	68	60	72	71.2	10.3537	4.63033
21	96	99	94	97	90	95.2	3.420526	1.529706	4.5	60	87	60	60	70	67.4	11.7813	5.26878
21	98	93	94	98	99	96.4	2.701851	1.208305	4.5	62	60	62	60	69	62.6	3.71484	1.66132
21	99	90	94	92	98	94.6	3.847077	1.720465	4.6	61	66	61	60	60	61.6	2.50998	1.1225
21	98	99	98	93	99	97.4	2.50998	1.122497	4.9	60	60	60	70	60	62	4.47214	2
21	99	96	99	94	97	97	2.12132	0.948683	8.7	60	66	60	75	60	64.2	6.57267	2.93939
21	95	96	93	97	98	95.8	1.923538	0.860233	12.7	60	75	60	77	60	66.4	8.79204	3.93192
21	99	99	99	91	96	96.8	3.49285	1.56205	15.2	70	76	70	80	63	71.8	6.49615	2.90517
21	99	98	90	98	99	96.8	3.834058	1.714643	16.9	73	78	73	83	68	75	5.70088	2.54951
21	97	99	99	96	99	98	1.414214	0.632456	18.1	82	86	82	84	89	84.6	2.96648	1.32665
21	98	95	98	97	98	97.2	1.30384	0.583095	18.9	88	87	88	82	77	84.4	4.82701	2.1587
21	96	99	98	93	98	96.8	2.387467	1.067708	19.3	98	89	98	92	86	92.6	5.36656	2.4
21	99	99	99	94	90	96.2	4.086563	1.827567	19.6	97	88	97	94	88	92.8	4.54973	2.0347
21	99	97	95	98	99	97.6	1.67332	0.748331	19.8	99	89	99	92	92	94.2	4.54973	2.0347
21	98	98	93	94	96	95.8	2.280351	1.019804	20	99	90	99	88	94	94	5.04975	2.25832
21	98	96	99	99	97	97.8	1.30384	0.583095	20	99	92	99	98	95	96.6	3.04959	1.36382

21	90	99	91	98	97	95	4.1833	1.870829
21	99	99	99	91	96	96.8	3.49285	1.56205
21	96	98	99	94	95	96.4	2.073644	0.927362
21	94	90	90	96	99	93.8	3.898718	1.74356
21	96	99	98	90	98	96.2	3.63318	1.624808
21	97	96	96	97	98	96.8	0.83666	0.374166
21	97	94	96	95	99	96.2	1.923538	0.860233
21	96	96	93	98	97	96	1.870829	0.83666
21	95	97	92	95	92	94.2	2.167948	0.969536
	95.95	95.10	95.40	95.31	95.19	**95.39**		
	2.96	2.36	2.96	2.78	2.88	**0.33**		
	0.22	0.18	0.22	0.21	0.22	**0.15**		

19.9	97	91	97	81	92	91.6	6.54217	2.92575
17.6	95	89	95	87	81	89.4	5.89915	2.63818
14.8	88	88	88	84	85	86.6	1.94936	0.87178
12.2	84	87	84	76	80	82.2	4.26615	1.90788
9.8	82	89	82	81	77	82.2	4.32435	1.93391
8	78	90	78	76	86	81.6	6.0663	2.71293
6.4	80	89	80	72	84	81	6.245	2.79285
5.4	66	89	66	72	78	74.2	9.65401	4.31741
4.7	60	88	60	60	70	67.6	12.1984	5.45527
	79.77	81.57	81.46	79.36	77.06	**79.85**		
	13.66	9.38	11.70	11.08	12.22	**1.84**		
	1.03	0.71	0.88	0.84	0.92	**0.82**		
